# Global Trends in the Research Landscape and Development of Micro- and Nanoplastics

**DOI:** 10.3390/toxics14090830

**Published:** 2026-09-19

**Authors:** Yidan Luo, Xinying Wu, Wuhu Guo, Lele Ru, Ziyu Wei, Wending Huang, Wenhao Zhang, Evgeny Abakumov, Huoqing Xiao, Yuejin Chen, Jia Wang, Yanqi Liu, Hannan Ahmad Anjum, Sunlin Chi, Wei Liu, Xianchuan Xie

**Affiliations:** 1 Jiangxi Provincial Key Laboratory of Environmental Pollution Control, Jiangxi Academy of Eco-Environmental Sciences and Planning, Nanchang 330039, China; 2 Key Laboratory of Poyang Lake Environment and Resource Utilization, Engineering Research Center of Watershed Carbon Neutralization, Ministry of Education, School of Resource and Environment, Nanchang University, Nanchang 330031, China5811123038@email.ncu.edu.cn (Z.W.);; 3 Anhui Nanxin Environmental Protection Technology Co., Ltd., 10th Floor, Building 15, Life Science and Technology Park, No. 8 Huangguan Road, High-Tech Zone, Anqing 246005, China; 4 Department of Applied Ecology, Saint Petersburg State University, Saint Petersburg 199178, Russia; e_abakumov@mail.ru; 5 Analytical Research Laboratory Microplastics, Microplastics Research Center, A Yaroslav-the-Wise Novgorod State University, B. St. Petersburgskaya Str. 41, Veliky Novgorod 173003, Russia; 6 Jiangxi Key Laboratory of Watershed Soil and Water Conservation, Jiangxi Academy of Water Science and Engineering, Nanchang 330029, China

**Keywords:** micro- and nanoplastics (MNPs), environmental fate, toxicological effects, global research evolution, human health risks

## Abstract

Over the past two decades, the development trajectory of micro- and nanoplastics (MNPs) research has been complex, with scattered focuses. A systematic review of global research trends is essential to clarify historical developments, identify key hotspots, and highlight emerging frontiers. To this end, we employed a thematic progression strategy combined with co-citation and keyword clustering networks to systematically map research hotspots and trace global progress on MNPs over the past five decades. A total of 12,851 related publications were extracted. Overall, the development of MNPs research demonstrates significant phases, diversity, and complexity, covering multiple dimensions including sources, environmental distribution, toxicological effects, tracing mechanisms, and management strategies. The research trajectory of MNPs clearly reveals a full-chain research path, from source identification and migration-diffusion to environmental interaction behaviors, highlighting the multi-media coupling propagation mechanisms and the potential risks of complex ecological disruptions. Studies on the distribution of MNPs in environmental media have evolved from early marine pollution concerns to a more integrated research field that covers water-soil-air media, multi-scale, and multi-morphological pollution behaviors, exhibiting high interdisciplinary and evolutionary features. Research on the toxicological effects of MNPs has evolved from “ecological biological responses” to “molecular mechanism identification” and then to “human health risks,” establishing a complete logical chain in toxicity understanding from macro to micro and from experimental studies to real-world exposure. These findings provide a foundation for understanding the environmental fate and multi-dimensional harm mechanisms of MNPs, offering references for institutions and researchers in future research directions.

## 1. Introduction

Since the large-scale production of plastics began in the 1950s, it has rapidly infiltrated core sectors such as packaging, construction, and healthcare due to its advantages of being lightweight, durable, and low-cost [1], becoming a symbol of modern industrial civilization. According to the Organization for Economic Co-operation and Development (OECD), by 2022, approximately 430 million tons of plastic were produced globally each year, with more than two-thirds of this being single-use products, which quickly become plastic waste after use [2]. If current production and consumption patterns continue, plastic production is projected to triple by 2060 [2]. As a result, with the proliferation of single-use plastic products, the issue of plastic pollution is becoming increasingly severe. Of particular concern is that in many developing countries, only 8% of plastic waste is recycled, while the remaining portion is either landfilled or incinerated, with approximately 76% of the waste directly leaking into the environment [3].

Micro- and nanoplastics (MNPs) derived from ubiquitous anthropogenic sources enter environmental systems, posing potential threats to the atmosphere, terrestrial soils, freshwater, marine ecosystems, biological organisms, and human health [4,5]. It is now well established that microplastics (MPs) exhibit global-scale multiphase pollution characteristics [6]. MNPs have been detected across these environmental compartments at varying concentrations worldwide [7,8], including remote and extreme environments such as Antarctica, Mount Everest, and the Mariana Trench [9,10,11]. They are also widely documented in plant and animal tissues [12,13], as well as in human organs and biological samples [14,15], indicating persistent ecological and health impacts.

The presence of plastic particles in the marine environment was first reported in 1972 along the coast of New England, USA, and within the Sargasso Sea [16]. In the same year, the Convention on the Prevention of Marine Pollution by Dumping of Wastes and Other Matter (London Dumping Convention) urged member states to implement measures preventing the disposal of plastic waste into the ocean [17]. Subsequently, the Basel Convention, adopted in 1989, incorporated plastic waste into a global regulatory framework to control its transboundary movement and management [18]. Nevertheless, substantial quantities of plastic waste continue to enter the environment due to individual behaviors and systemic mismanagement, such as littering and incomplete waste collection, facilitating its transport through terrestrial and aquatic pathways [19]. As plastic pollution became increasingly severe, Thompson et al. [20] conducted a seminal study on plastic debris from beaches, estuarine sediments, and subtidal deposits around Plymouth, UK, leading to the formal introduction of the term “microplastics,” which marked the beginning of focused scientific investigation into this pollutant.

In the past decade, research on microplastics has developed rapidly. In 2014, only 20 papers containing the keyword “microplastics” were indexed in Scopus, the world’s largest abstract and citation database. By 2024, this number had grown to nearly 6000. Over the past two decades, research on MNPs has experienced exponential growth. The history and focus of this research field are continuously expanding, which has led to shifts in research trends and hotspots, with prominent contributing institutions and individuals still to be clearly identified. More critically, the field’s development is complex and fragmented, lacking sufficiently focused and systematic research. This has resulted in an unclear evolutionary path for the discipline, with future research priorities and directions remaining highly dispersed and uncertain. Given the enormous volume of related studies, it is essential to conduct a dedicated review and analysis of trends in this knowledge domain, uncovering the historical evolution of key hotspots, summarizing emerging trends in MNPs research, and identifying the future directions of international research. Such an analysis would help clarify the development trajectory of the field and guide future research. Particularly, while numerous inductive and critical reviews on MNPs research have been published, these are often focused on specific topics, and a global perspective on hotspot tracking is urgently needed [21].

Bibliometric analysis utilizes visualization tools to examine a large volume of published academic literature. Compared to traditional research methods, it offers distinct advantages in tracking research hotspots and the evolutionary history of a field [22]. To date, no studies have analyzed the global research trends of MNPs from a bibliometric perspective. Only a few studies have reported step-by-step guides for scientometric analysis focused on MNPs research [23]. Therefore, all reviews in this study were performed in accordance with the PRISMA process [24]. The collected papers can, to some extent, reflect trends at the scientific frontier promptly, and through bibliometric methods, we can perform statistical analysis and content mining. Our primary goal is to systematically chart the temporal evolution of MNPs research, using co-citation reference networks and co-occurrence keyword networks to identify the evolution of key research themes. Our secondary goal is to provide researchers with metrics for research networks (countries, institutions, authors, and journals) and identify the richness, gaps, emerging trends, biases, and limitations of the research. This analysis will provide valuable insights into the development trajectory of the highly globalized and emerging field of MNPs.

## 2. Data Acquisition and Visualization Analysis

**Figure 1:**
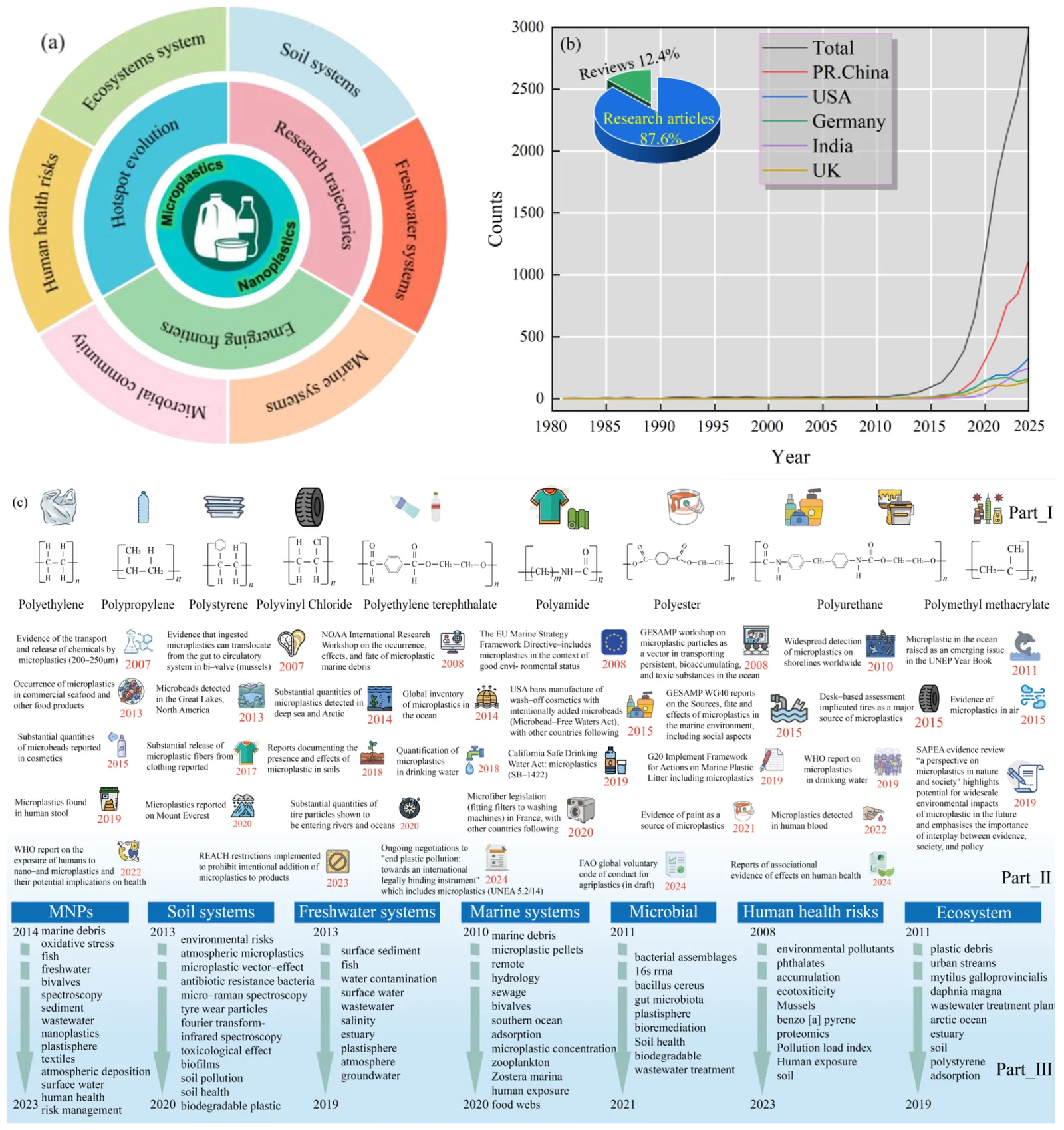
(**a**) Strategy for the search and analysis of MNPs research. (**b**) Global trend of publications and the changing consistency of the annual publication quantities in the top five countries/regions from 1981 to 2022, including a world map based on the total publications of different countries/regions. (**c**) The phased evolution of MNPs research: Part I—Plastic products and chemical formulations; Part II—Key developmental milestones in microplastics research (adapted from Thompson et al. [6]); Part III—Evolution of research hotspots under a thematic progression strategy.

We conducted a comprehensive search of the Web of Science Core Collection (WoSCC), which is recognized as the most inclusive database for bibliometric analysis [25]. Our search strategy incorporated both thematic terms and keywords related to MNPs. The core query for MNPs was defined as TS = (“microplastics”) OR (“nanoplastics”). Based on this core theme, subtopics were identified using targeted search terms related to “marine,” “freshwater,” “soil,” “toxicity,” “microorganisms,” “human,” and “ecosystem” (Figure 1a). The complete search strings are provided in the Supplementary Text S1. The data source was limited to the Science Citation Index Expanded, without restriction on document type, language, or publication year. Articles formally published before 28 February 2025 were included. Complete records and cited references were exported from WOSCC as plain text files with tab-delimited format. Duplicate entries were removed using CiteSpace (Version 6.3.R1.) before visualization analysis (Text S2). Detailed analysis steps, parameter settings, and classification criteria for the visualization outputs are available in the Supplementary Information (Texts S2–S4).

## 3. Advances in MNPs Research

From 1981 to 2025, a total of 12,851 publications related to microplastics were indexed in the Web of Science (WoS) database. Among these, 10,818 were original research articles (84.2 percent), 1529 were review papers (11.9 percent), and the remainder consisted of conference proceedings and other document types (Figure 1b). The annual number of publications on microplastics has shown a marked upward trend. In 2016, only 135 articles were published, increasing to 236 in 2017, followed by a consistent annual growth rate of no less than 15 percent. In 2020, the number of publications exceeded 1000 (1172 articles), reaching 2450 in 2023 and approaching 3000 in 2024 (2944 articles) (Figure 1b). This trend highlights that microplastics, as an emerging class of environmental contaminants, have become a central topic in the global environmental science community in recent years (Figure 1c).

In terms of contributions by country or region, the top ten countries collectively produced 11,036 publications, accounting for 85.88 percent of the total. China leads in scientific output, with institutions such as the Chinese Academy of Sciences and the University of Chinese Academy of Sciences contributing a total of 4044 publications, representing more than 31.47 percent. The United States (1396 articles, 10.86 percent), Germany (1034 articles, 8.05 percent), and India (827 articles, 6.44 percent) followed (Figure 1b, Figures S1–S4 and Table S1). Notably, the United Kingdom ranks fifth in total article count (TA 746, 5.80 percent); it ranks second in total citations (TC 93,461), just after China (TC 181,923), and has the highest citations per article (CPA 125). Its h-index (143) is also second only to China (198) and the United States (150) (Table S1, Figures S5 and S6).

Over 200 authors have been recorded as contributors to microplastic research (Figure S7). Koelmans et al. recorded the highest number of publications from Wageningen University and Research Centre (TA 232, TC 28,222, CPA 121.64, H-index 79). This was followed by Rochman et al. from the University of Toronto (TA 140, TC 17,880, CPA 127.71, H-index 53) and Shi Huahong from East China Normal University (TA 152, TC 18,787, CPA 123.60, H-index 67). Table S3 and Figures S8–S11 present information on the top 10 authors who have made the most significant contributions to publications in this field. Among these authors, five are from China, and two are from Germany. Notably, Thompson et al. from Imperial College London ranks fourth in terms of publication volume. Still, he leads both in CPA (146.35) and TC, indicating the high academic value and impact of his research.

An analysis of the top ten journals by publication volume reveals that microplastic research predominantly focuses on inflammation and marine debris (Table S2 and Figure S12). Forty percent of the articles in this field are published in environmental science journals, which is substantially higher than the proportions in biology, toxicology, and water resources fields (Tables S4 and S5). The Web of Science (WoS) database covers 175 subject categories, with the top five being Environmental Science (38.05%), Environmental Engineering (9.11%), Marine and Freshwater Biology (7.97%), Toxicology (3.99%), and Water Resources (3.99%), which together account for 63.12% of the total publications.

## 4. Evolution of Research and Key Hotspots in MNPs Studies

### 4.1. Global Research Trajectories in the Field of MNPs

#### 4.1.1. Origins of MNPs Research

**Figure 2:**
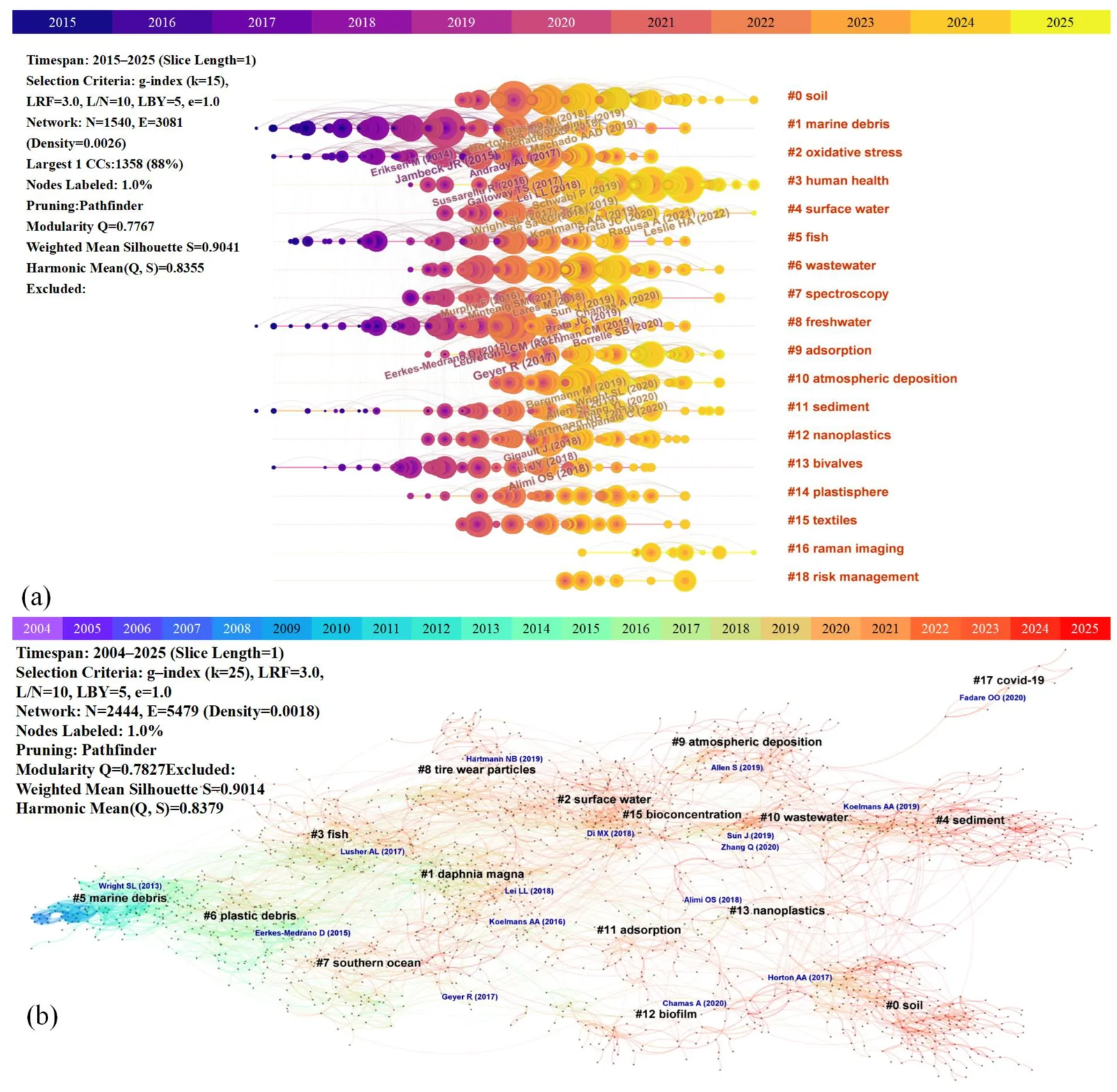

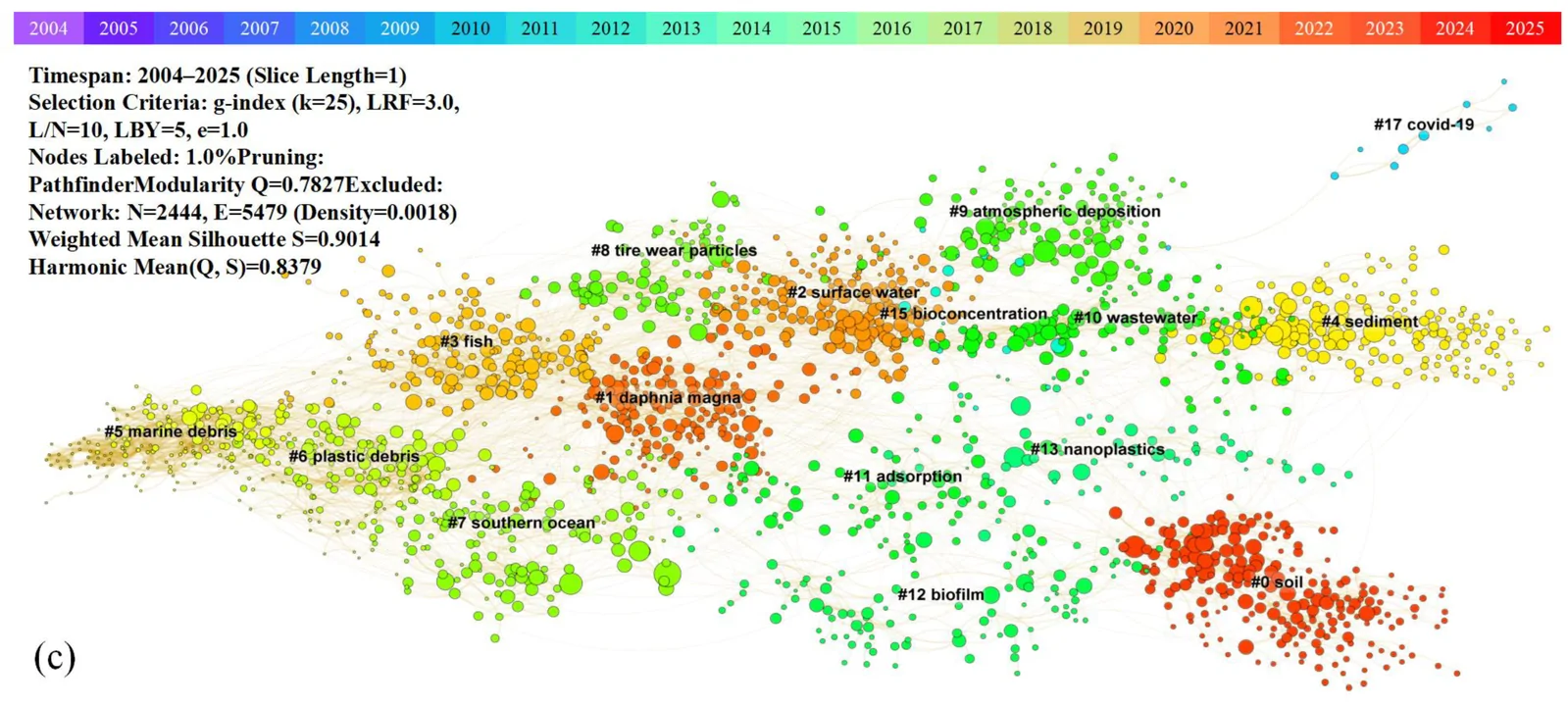
Co-citation references network (1981–2021) and correspondent clustering analysis obtained using CiteSpace. (**a**) Timeline visualization of keyword networks; (**b**,**c**) Co-citation reference network with cluster visualization and burstiness of hotspots; The size of a node (article) is proportional to the number of times the article has been co-cited; burstiness is represented by red tree rings, with either important citation burst.

To systematically elucidate the knowledge structure and evolutionary trajectory of research on MNPs, we constructed a co-citation reference network. Within this network, 18 thematic clusters were identified. The modularity (Q = 0.7767) and silhouette score (S = 0.9041) of the network both indicate a high degree of structural rationality and clustering validity, suggesting that these clusters are well-defined and cohesive (Figure 2a and Figures S13 and S14). Further nebula diagram analysis revealed four major research evolution trends in the MNPs field, reflecting the dynamic expansion of research themes and interdisciplinary integration across different stages of development.

Although the term “microplastics” was formally introduced by Thompson et al. in 2004 [20], scientific attention to plastic particle pollution in the marine environment dates back to earlier decades. As early as 1972, Edward J. Carpenter and his research team published the first report on the widespread distribution of plastic particles in the offshore waters of New England and the Sargasso Sea in Science, laying the foundation for marine plastic pollution research [16]. In 1997, Moore discovered a massive accumulation of floating plastic debris in the North Pacific Subtropical Gyre. This finding, later reported in 2001, marked a critical turning point in drawing global attention to marine plastic pollution [26].

Research on MNPs has gradually emerged over the past two decades, with a notable increase in activity following the introduction of the term “microplastics” in 2004. During the decade from 2004 to 2014, the number of related studies was relatively limited, representing an early stage of development (Figure 1b). Around 2015, the field experienced rapid growth, marked by an exponential rise in the volume of publications and an increasing diversification of research topics. Overall, the development of MNPs research has demonstrated significant phases, diversity, and complexity, covering various dimensions such as environmental distribution, toxicological effects, source mechanisms, and management strategies (Figure 1b and Figure 2b).

The scientific definition of nanoplastics (NPs) emerged later. In 2011, the European Commission first formally defined nanomaterials in its “Commission Recommendation of 18 October 2011 on the definition of nanomaterial,” marking the beginning of systematic research on NPs. NPs are smaller particles formed through the physical degradation of plastics in the natural environment, aided by processes such as microbial action. They possess a higher specific surface area and hydrophobicity, allowing them to adsorb pollutants such as heavy metals, persistent organic pollutants (POPs), and antibiotics, which enhances their environmental persistence and composite toxicity [27]. Research on NPs is gradually establishing itself as an independent hotspot within the MNPs field (Cluster #12, 53 articles, S = 0.907, 2018) (Figure 2b). Although NPs exhibit stronger mobility and greater bioavailability in ecosystems, their concentration and distribution in environmental media remain difficult to accurately assess due to limitations in detection and quantification methods. Currently, the potential risks posed by NPs in freshwater, soil, and marine systems are beginning to gain attention. Studies indicate that NPs can penetrate cellular membranes [28] and even biological barriers [29], affecting microbial communities [30], enzyme activities, and various physiological functions [31,32,33]. The health and ecological issues raised by NPs may surpass those of traditional microplastics. With the advancement of highly sensitive detection technologies, NPs are expected to become a prominent frontier in environmental science research in the future.

#### 4.1.2. Evolution of Research Hotspots on the Distribution and Migration of MNPs

In the co-citation network we constructed, the first significant research direction focuses on the distribution characteristics and pollution status of MNPs in various environmental media. The literature associated with relevant clusters demonstrates a high degree of structural consistency and disciplinary focus, including Cluster #1 (“Marine Litter”, 144 articles, S = 0.835, 2014), Cluster #8 (“Freshwater”, 78 articles, S = 0.876, 2016), Cluster #4 (“Surface Water”, 104 articles, S = 0.893, 2019), Cluster #0 (“Soil”, 159 articles, S = 0.948, 2019), and Cluster #10 (“Atmospheric Deposition”, 62 articles, S = 0.974, 2020). These clusters collectively outline the temporal evolution and shifting research emphasis in the study of MNPs distribution across environmental media (Figure 2a,c).

Early MNPs research began with the marine system, driven by concerns over plastic pollution in the oceans. As related studies deepened, scientists gradually expanded their focus from the oceans to terrestrial ecosystems and the atmosphere, exploring the environmental behavior and fate of MNPs in surface waters, soils, sediments, and the atmosphere. Due to various mechanisms such as physical weathering, ultraviolet radiation, and biological activity that continuously fragment plastics in the environment [34], forming particles at the micron or even nanometer scale [35], these particles exhibit significantly enhanced mobility across different environmental media, particularly being able to suspend and settle in the air, leading to a vast distribution range. In marine systems, MNPs have been found in the surface ocean, deep sea, polar regions, and nearshore areas. They have even been detected at the deepest point of the Mariana Trench, the Challenger Deep, with up to 71.1 microplastic particles·kg^−1^ of dry sediment [36,37,38]. Benthic organisms and pelagic ecosystems are commonly affected, showing clear accumulation and potential ecological toxicity risks.

Microplastic pollution in soil systems has become globally widespread, with particularly significant concentrations in agricultural areas, around landfills, and in regions with frequent industrial activities. Existing studies indicate that in Yunnan Province, China, the abundance of microplastics in soils of high tourist traffic areas ranges from 7.1 × 10^3^ to 4.3 × 10^4^ particles·kg^−1^ [39]; in agricultural soils in Switzerland, the dominant microplastics are PE, PS, and PVC, with an average abundance of 593 particles·kg^−1^ [40]; in some areas of Mexico, the abundance reaches up to 2770 particles·kg^−1^ [41]; in mangrove soils in northern Brazil, concentrations range from 3.1 × 10^3^ to 1.8 × 10^4^ particles·kg^−1^ [41]; and in urban soils of Sydney, Australia, the detected microplastics, mainly composed of PVC, PE, and PS, exhibit abundance levels ranging from 3.0 × 10^2^ to 6.7 × 10^4^ mg·kg^−1^, with particle sizes mostly smaller than 5 mm [42].

The widespread presence of MNPs in freshwater systems has also been confirmed. In Europe, the average microplastic concentration in the River Thames, UK, is approximately 0.028 particles·m^−3^ [43]; in Asia, the levels are more pronounced, with concentrations in eight lakes in Changsha, China, ranging from 2425 to 7050 particles·m^−3^ [44]. Furthermore, in various water bodies in China, including Honghu Lake in Hubei, Dongting Lake in Hunan, and the Pearl River in Guangdong, as well as in the Loddong River in South Korea and the Saigon River in Vietnam, microplastic concentrations range from 900 to 2800 particles·m^−3^, 1250 to 4650 particles·m^−3^, 379 to 7924 particles·m^−3^, 293 to 4760 particles·m^−3^, and 1.72 × 10^5^ to 4.19 × 10^5^ particles·m^−3^, respectively [45,46,47,48], indicating a high concentration load in water bodies across East Asia.

In recent years, research on MNPs has gradually extended to the atmosphere. Liao et al. [49] found that the particle size of microplastics in indoor and outdoor air samples collected in Wenzhou, China, was primarily concentrated in the 0–30 μm range, with estimated average particle sizes of 107 μm and 71 μm, respectively. Roblin et al. [50] observed that approximately 15% of the particles in dry and wet deposition samples collected from four sampling sites in Ireland were microplastics, with particle sizes concentrated in the 200–400 μm range, and an average size reaching 640 μm. These findings suggest that airborne transport could serve as an essential pathway for the long-range migration of MNPs, and the potential exposure risk in indoor air quality should not be overlooked. It is important to note that the degradation period of plastic materials in the natural environment often spans centuries, especially in cold, low-microbial-activity environments such as polar regions and deep-sea areas, where their environmental lifespan may be even longer [51]. This persistence further exacerbates the ecological risks associated with the continuous accumulation of MNPs across various environmental media.

#### 4.1.3. Research Progress on the Sources and Environmental Behaviors of MNPs

Co-citation map analysis indicates that the identification of MNPs sources and their environmental behavior constitutes the third major research trend in this field (Figure 2a). This trend focuses on how MNPs enter ecosystems, their migration and fate in various environmental media, and their complex interactions with environmental factors.

In terms of origin, MNPs primarily enter the environment in two forms: primary microplastics and secondary microplastics. Primary MNPs refer to microplastic particles that are directly manufactured during industrial production, commonly found in products such as cosmetics, plastic coatings, packaging materials, and electronic goods. Secondary MNPs, on the other hand, result from the degradation of larger plastic items due to natural or anthropogenic forces, with primary sources including the aging and fragmentation of agricultural plastic films, degradation of single-use plastic packaging, and solid waste treatment methods such as incineration or landfill [52,53].

Among the various pathways of input, wastewater treatment plant effluents are considered one of the most significant routes for MNPs entering aquatic environments [54], corresponding to cluster #6 (“Wastewater,” 87 papers, S = 0.961, 2018). Although modern wastewater treatment processes can remove some particulate pollutants, MNPs, due to their small size and low density, are often not completely captured, and they are discharged into rivers, lakes, and other water bodies with treated water [55]. Additionally, the abrasion of synthetic textile fibers, wear of synthetic rubber tires, and urban dust are significant sources of atmospheric microplastics [56], corresponding to cluster #15 (“Textiles,” 22 papers, S = 0.975, 2018). These two clusters share research hotspots and reveal significant sources of MNPs in urban environments.

In soil media, MNP contamination is often associated with the long-term accumulation of discarded plastic packaging. Environmental factors such as ultraviolet radiation, microbial degradation, and physical abrasion can accelerate the fragmentation process [34]. Studies have shown that approximately 80% of microplastics in marine systems originate from land-based inputs, including plastic debris carried by rivers and shoreline litter, with only about 20% stemming from marine activities, primarily commercial fishing gear that has been abandoned [57].

Surface sediments in deep-sea regions are considered a significant final repository for microplastics, exhibiting distinct characteristics of storage and migration [58]. This corresponds to cluster #11 (“Sediments,” 56 papers, S = 0.859, 2017). Within this cluster, numerous studies focus on the abundance, particle size distribution, composition, and vertical migration behavior of microplastics (MPs) in various types of sediments, reflecting their complex migration pathways on a geographical scale.

Furthermore, the surface of microplastics, acting as an exogenous hydrophobic carrier, provides a novel ecological niche for microorganisms, leading to the formation of a unique ecosystem centered around microorganisms—referred to as the “plastisphere” [59]. This corresponds to cluster #14 (“Plastisphere,” 28 papers, S = 0.977, 2018). Research indicates that the surface of microplastics is prone to accumulating pathogenic bacteria, antibiotic-resistant bacteria, and functional microorganisms, posing a potential risk for disease transmission. These particles could also serve as vectors for the spread of “superbugs,” thereby increasing the complex threat to ecosystems and human health [60,61].

#### 4.1.4. Synergistic Evolution of Detection Technologies and Risk Management

With the deepening of MNPs research, innovations in detection and identification technologies have become a key driver for the advancement of the field, constituting the fourth major research trend. This trend is primarily represented by cluster #7 (“Spectroscopy,” 80 papers, S = 0.882, 2019) and cluster #16 (“Raman Imaging,” 13 papers, S = 0.992, 2021), highlighting the continuous breakthroughs in methods for characterizing MNPs. MNPs detection includes two main categories: physical characterization and chemical analysis methods. Early methods, relying on visual identification, were significantly limited, particularly in terms of accuracy under conditions of reduced particle size [62]. Subsequently, methods based on spectral analysis rapidly developed, such as Fourier-transform infrared (FTIR) spectroscopy, which is suitable for the study of polymorphic systems. To this day, this method remains one of the most widely used in microplastic research and has proven effective in identifying, quantifying, and characterizing microplastics in aquatic environments [63], terrestrial environments [5,10], atmospheric environments [64,65], as well as in drinking water [66], food [67], and even biological samples [68]. Raman spectroscopy, a scattering technique, is a non-destructive analytical method for microplastics, with a minimum detectable plastic size of 1 μm [69]. However, Raman spectroscopy has significant drawbacks, particularly fluorescence interference, especially when analyzing environmental samples. Inorganic substances (such as clay minerals, dust particles), organic compounds (such as humic substances), biological impurities [70], and additives (such as dyes) [71] in the sample matrix can induce fluorescence interference. Therefore, sample preparation requirements are higher for environmental samples analyzed using Raman spectroscopy.

Notably, it should be pointed out that nanoplastics-associated clusters observed before 2018 originate from conceptual keyword indexing in publications rather than practical instrumental measurements. Although FTIR and Micro-Raman spectroscopy are constrained by their physical resolution limits for real-world nanoplastic detection, many pre-2018 papers discussed the theoretical fate and potential risks of nanoplastics and labeled “nanoplastics” as keywords in titles or abstracts, which led CiteSpace to assign these documents to nanoplastics-relevant clusters regardless of actual experimental particle measurement.

Building on the accumulation of research on the ecological and health effects of microplastics, risk management research has gradually emerged, corresponding to cluster #18 (“Risk Management,” 6 papers, S = 0.999, 2020). Studies have shown that MNPs may trigger adverse health effects such as inflammation, endocrine disruption, and cellular damage, leading to increased regulatory concern globally. The European Union has successively introduced the “Microplastics Strategic Action Plan” (2021) and the “Microplastics Restriction Regulation” (2023), driving the integration of MNPs into environmental management frameworks. However, during the fifth negotiation meeting of the “End Plastic Pollution Resolution” at the end of 2024, although some issues reached consensus, no legally binding international agreement has been established. This indicates that global governance of MNPs is still in the early stages of policy negotiation and regulatory coordination.

### 4.2. Evolution of Research Hotspots on MNPs in Different Environmental Matrices or Subfields

#### 4.2.1. Evolution of MNPs Research in Terrestrial Pollution and Soil Systems

**Figure 3:**
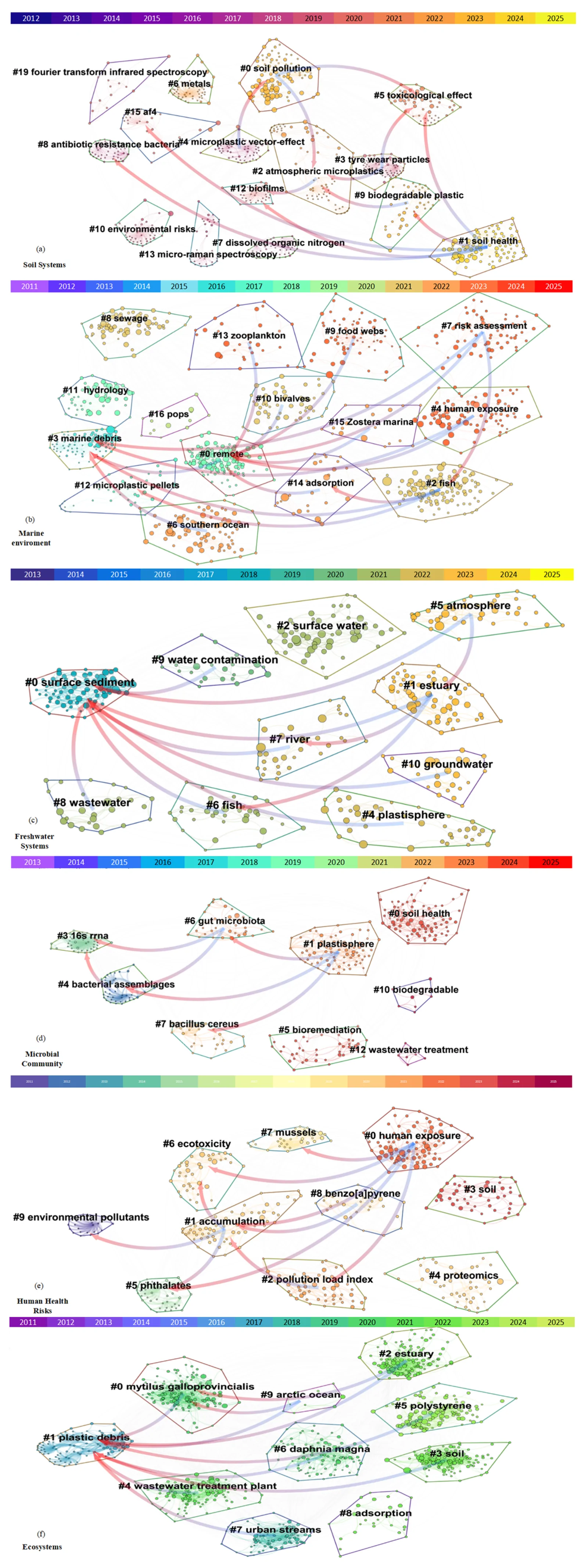
Shifts in research hotspots across subtopics. The first panel shows the results of keyword clustering analysis over time; the second panel shows the timeline visualization of keyword networks (**a**): soil systems; (**b**): freshwater systems; (**c**): marine environment; (**d**): microbial community; (**e**): human health risks; (**f**): ecosystems.

Land is the primary source and potential sink of microplastics, particularly from the perspective of atmospheric migration of microplastics. As research on microplastics in marine environments has matured, the soil system has gradually emerged as a frontier in the field of “microplastics and nanoplastics,” becoming one of the eight core subtopics, including “soil, ocean, freshwater, human, animals, ecology, toxicity, and microorganisms.” Based on a bibliometric co-citation network analysis (Q = 0.8285, S = 0.9152), research on soil microplastics has shown a clear evolutionary trajectory (Figure 3a).

Early studies focused on the sources of microplastics in soil, particularly clusters #2 (“Atmospheric Microplastics”; 71 papers; S = 0.913; 2014) and #3 (“Tire Wear Particles”; 70 papers; S = 0.977; 2016). Atmospheric transport (2008–2021) and tire wear were identified as significant input pathways, with particles migrating to remote areas through deposition and wind action [58]. Subsequent studies shifted toward detection and quantification methods for soil microplastics, forming clusters #13 (“Micro-Raman Spectroscopy”; 36 papers; S = 0.986; 2015), #15 (“Asymmetric Flow Field-Flow Fractionation”; 34 papers; S = 0.951; 2016), and #19 (“Fourier Transform Infrared Spectroscopy”; 15 papers; S = 0.957; 2016). FTIR and Raman spectroscopy became central techniques for identifying the types, morphologies, and particle sizes of microplastics in soil.

As research progressed, two directions emerged. On one hand, clusters #4 (“Microplastic Carrier Effect”; 62 papers; S = 0.854; 2015), #6 (“Metals”; 58 papers; S = 0.993; 2016), and #7 (“Dissolved Organic Nitrogen”; 54 papers; S = 0.974; 2015) focused on the impact of microplastics on soil physicochemical properties and nutrient cycling, with research concentrated between 2015 and 2020. On the other hand, clusters #0 (“Soil Pollution”; 97 papers; S = 0.788; 2020), #1 (“Soil Health”; 95 papers; S = 0.867; 2020), #5 (“Toxicological Effects”; 61 papers; S = 0.892; 2017), #10 (“Environmental Risks”; 46 papers; S = 0.890; 2013), and #12 (“Biofilms”; 36 papers; S = 0.955; 2017) explored the composite pollution and ecosystem disruption caused by MNPs. Studies have shown that MPs can alter soil pore structure, moisture retention, bulk density, and the content of elements such as organic carbon, total nitrogen, total phosphorus, and potassium, thereby reducing soil fertility [39,72,73]. Additionally, they exhibit significant adsorption capacity for polychlorinated biphenyls, pesticides, heavy metals, and other contaminants, forming potential pollutant complexes [74].

In the ecological dimension, microplastics exhibit multiple toxicological effects on soil organisms, such as earthworms, springtails, and snails, including intestinal damage, immune dysregulation, reproductive inhibition, and disruption of microbial communities [75,76,77,78]. Additionally, microplastics can be transferred through the food chain, impacting higher trophic level organisms. According to a study by Huerta Lwanga et al. [41], microplastics in the soil, in addition to being ingested by earthworms, were found to accumulate in chicken manure with a dry-weight-based concentration ratio as high as 10^5^. It should be noted that this value represents a dry-weight-derived concentration ratio rather than a standardized bioaccumulation kinetic factor (BAF). Microplastics were also detected in the organs of chickens. This indicates that microplastics may accumulate through the food chain, thereby posing a threat to human health. In plants, MPs hinder water and nutrient absorption by roots, suppressing nutrient metabolism and growth [79,80]. Regarding soil microorganisms, MPs form a “plastic interface” microecology, significantly altering bacterial community structure and functional metabolism [81]. While certain MPs may promote microbial activity [82], the overall ecological risks remain a significant concern.

With the promotion of biodegradable plastics, cluster #9 (“Biodegradable Microplastics”; 49 papers; S = 0.899; 2021) indicates a shift in research focus towards mitigation strategies (Figure 3a). However, the rapid degradation of biodegradable plastics in soil could exacerbate short-term exposure to microplastics [83,84], leading to new environmental challenges. Currently, research on the distribution, behavior, and fate of microplastics in soil remains insufficient due to limitations in the standardization of detection techniques and the complexity of soil ecosystems [85]. There is an urgent need for standardized monitoring methods and risk assessment frameworks to support future management and remediation strategies.

#### 4.2.2. Evolution of Microplastic Pollution Research and Hotspot Focus on Terrestrial Freshwater Systems

Based on co-citation network analysis of the literature (Q = 0.6416, S = 0.8463), three major research trends were identified in the network, reflecting the systematic understanding of the pollution mechanisms and ecological risks of microplastics in terrestrial freshwater environments (Figure 3b). As research on marine microplastics has matured, the research focus has gradually shifted towards terrestrial environments. Rivers, as a key conduit for the transfer of microplastics from land to the ocean, have garnered significant attention in terms of their ecological processes and environmental exposure.

Early studies focused on the abundance and distribution of MPs in inland riverbank sediments, corresponding to Cluster #0 (“Surface Sediments”; 78; S = 0.921; 2013), which laid the foundation for subsequent research (Figure 3b). Over time, studies expanded to the identification of sources of microplastics in terrestrial freshwater systems, resulting in the formation of Clusters #5 (“Atmospheric Transport”; 37; S = 0.818; 2019) and #8 (“Wastewater”; 26; S = 0.893; 2017). Research has revealed that atmospheric deposition can transport plastic particles to remote inland areas [86], while household waste, industrial emissions, personal care products, and synthetic fiber abrasion are the primary input sources [87]. Although wastewater treatment systems can intercept most microplastics, residual particles still enter freshwater systems through discharge [55]. Plastics in the environment degrade into microplastics under the influence of photodegradation and biodegradation, which, through processes such as rainfall, runoff, and atmospheric deposition, enter water bodies and sediments, resulting in persistent pollution [88].

Research has further focused on the abundance of microplastics in various types of freshwater bodies, primarily including Cluster #2 (“Surface Water”; 56; S = 0.853; 2017), Cluster #10 (“Groundwater”; 24; S = 0.892; 2016), Cluster #7 (“Rivers”; 34; S = 0.820; 2018), and Cluster #1 (“Estuaries”; 59; S = 0.590; 2019) (Figure 3b). Studies have shown that microplastic pollution exists at varying degrees in rivers, lakes, groundwater, and drinking water [89,90,91,92]. Notably, in urban rivers, microplastic concentrations are significantly higher than in nearshore marine environments [93], leading to widespread public concern about the risks of human ingestion.

The third category of studies focuses on the ecological toxicity and composite risks of microplastics, corresponding to Cluster #4 (“Plastic Interactions”; 41; S = 0.847; 2019), Cluster #6 (“Fish”; 34; S = 0.772; 2016), and Cluster #9 (“Water Pollution”; 25; S = 0.911; 2016). Research indicates that microplastics can accumulate in various aquatic organisms, including fish [94], benthic organisms [95], mussels [96], and algae [97], causing damage to tissues and physiological disruptions. The toxicity of microplastics is exacerbated by the adsorbed toxic additives (such as polybrominated diphenyl ethers and bisphenol A) and external pollutants (such as hydrophobic organic compounds and heavy metals), creating significant composite pollution effects [98]. Furthermore, as freshwater systems are a primary source of water for humans, the potential threat of their contamination to human health has garnered increased attention. Research on microplastics has predominantly focused on animal subjects, with studies on humans still in the preliminary stages [99]. Although existing research has revealed pathways for human exposure to microplastics and initial levels of exposure [100], systematic studies on their metabolic mechanisms and health consequences remain lacking, necessitating interdisciplinary collaborative efforts.

#### 4.2.3. Research Evolution of MNPs in the Marine Environment

Research on MNPs originated in marine environments, and the in-depth studies conducted during this phase laid a solid foundation for subsequent research on MNPs in terrestrial ecosystems (Figure 2a). Based on bibliometric co-citation network analysis (Q = 0.6613, S = 0.8266), research on marine MNPs has demonstrated two major development trends (Figure 3c). The first trend focuses on the sources, migration, and distribution of marine plastic debris. Initially, it centered on Cluster #3 (“Marine Debris”; 87 articles; S = 0.935; 2010), gradually evolving to include Cluster #0 (“Remote Areas”; 115 articles; S = 0.726; 2014), Cluster #11 (“Identification”; 56 articles; S = 0.839; 2014), Cluster #6 (“Southern Ocean”; 78 articles; S = 0.837; 2018), and Cluster #8 (“Sewage”; 68 articles; S = 0.767; 2017). The sources of marine plastic pollution are complex, with approximately 80% being land-based inputs, followed by plastic generated from maritime activities [101]. Plastic waste can enter the oceans directly via rivers and wastewater systems or be transported indirectly through water cycles [102]. Microplastics are widely distributed across global oceans, from the poles [103] to the equator, extending from densely populated areas to remote, uninhabited islands, indicating their global and pervasive nature.

The second trend focuses on the ecological impacts and potential risks of microplastics, encompassing Cluster #12 (“Microplastic Particles”; 42 articles; S = 0.909; 2012), Cluster #16 (“Persistent Pollutants”; 13 articles; S = 0.955; 2015), and Cluster #14 (“Adsorption”; 32 articles; S = 0.912; 2018). It then evolved into Cluster #2 (“Fish”; 103 articles; S = 0.824; 2017), Cluster #10 (“Bivalves”; 56 articles; S = 0.792; 2017), and Cluster #9 (“Food Web”; 67 articles; S = 0.767; 2017), reflecting a shift in the focus of research towards biological toxicity (Figure 3c. The impact of microplastics on marine organisms is primarily manifested in the physical obstruction of the respiratory and digestive systems after ingestion, as well as toxic effects caused by the microplastics themselves and the toxic substances they adsorb (such as polybrominated diphenyl ethers, bisphenol A, and other additives, as well as hydrophobic organic pollutants and heavy metals) [104]. Furthermore, microplastics can accumulate in higher trophic level organisms through the food chain, posing a potential threat to human food security. Phytoplankton, as the foundation of marine ecosystems, is particularly vulnerable; disruption of their population structure could trigger broader ecological impacts. The current understanding of the effects of microplastics on phytoplankton remains controversial, with some studies indicating that microplastics inhibit algal growth [105,106,107], while others report opposite effects that promote growth [108,109]. Related clusters include #13 (“Zooplankton”; 35 articles; S = 0.875; 2019) and #15 (“Macrophytes”; 18 articles; S = 0.941; 2019).

#### 4.2.4. Research Progress on the Microbial Community on the Surface of Microplastics and Its Ecological Functions

Microplastics have been recognized as a novel microbial habitat, termed the “plasticphere” [59], influencing microbial community structures and element cycling in various environmental media. Based on a bibliometric co-citation network analysis (Q = 0.7435, S = 0.8845), research on the microbial communities residing on microplastic surfaces has emerged as a significant focus, with each research direction being relatively independent (Figure 3d). Early studies primarily concentrated on the biological communities attached to microplastic surfaces, particularly focusing on Cluster #3 (“16S rRNA”; 46 publications; S = 0.946; 2012) and Cluster #4 (“Bacterial aggregates”; 45 publications; S = 0.665; 2011). Research on marine plastics and attached microorganisms began in the 1970s [16]. Using 16S rRNA gene sequencing, scientists identified microorganisms colonizing microplastic surfaces and discovered dynamic succession in microbial communities [110]. Since plastics are synthetic polymers derived from petroleum, coal, and natural gas, some heterotrophic microorganisms can utilize them as a carbon source, leading to microplastic degradation. For example, *Pseudomonas* sp. and *Rhodococcus* sp. can degrade polypropylene [111], corresponding to Cluster #7 (“Bacillus cereus”; 34 publications; S = 0.941; 2016). Simultaneously, Cluster #6 (“Gut microbiota”; 34 publications; S = 0.941; 2017) focused on the effects of microplastics on animal gut microbiomes. Oral ingestion is the primary route of human microplastic exposure, with the gastrointestinal tract being the main accumulation and toxicity target [112]. Studies have shown that microplastics accumulate in the gut and disrupt the composition and function of the microbiota [113,114], leading to dysbiosis, which, through pathways such as lipid metabolism, bile acid metabolism, and neurotransmitter metabolism, can cause health damage to vital organs like the liver and brain [115,116].

Clusters #4, #6, and #7 further evolved into Cluster #1 (“Plastisphere”; 77 publications; S = 0.826; 2018), a term that describes the complex ecosystem of plastic matrices and their attached heterotrophic, autotrophic, and symbiotic communities. Studies have found pathogenic bacteria within the marine plastisphere, which can be spread by ocean currents to other regions, leading to microbial invasion [117] and posing potential ecological threats.

Moreover, there are several relatively independent research clusters. Cluster #10 (“Biodegradable”; 9 publications; S = 0.966; 2020) and Cluster #5 (“Bioremediation”; 42 publications; S = 0.861; 2019) primarily explore the degradation potential of microorganisms such as actinomycetes, algae, bacteria, and fungi on microplastics, emphasizing the importance of developing biodegradation and recycling technologies [118,119]. Cluster #12 (“Wastewater treatment”; 6 publications; S = 0.994; 2021) focuses on the removal efficiency of microplastics in wastewater treatment processes. Although existing wastewater treatment facilities are not explicitly designed to eliminate microplastics, they can generally reduce the concentration of microplastics in the water phase effectively [120], with most microplastics being transferred to sludge, where they are not completely degraded [121,122,123], eventually entering soil environments through activated sludge. In recent years, advancements in biotechnology have led to the discovery of numerous plastic-degrading strains and enzymes, providing new avenues for addressing the biological degradation of microplastics in activated sludge [124].

Cluster #0 (“Soil Health”; 85 publications; S = 0.887; 2020) primarily focuses on the impact of microplastics on soil microbial communities. Research indicates that the microbial composition and dominant species on plastic surfaces fluctuate significantly over time, influenced by plastic properties and both biotic and abiotic environmental factors [125]. Microorganisms in the plasticphere play a key role in the weathering and fragmentation of plastics. Through microbial action, microplastics can degrade into submicron to nanometer-sized particles, which migrate in colloidal form and quickly deposit in groundwater, thereby increasing the risk of groundwater contamination and threatening drinking water safety [126]. However, Huang et al. [1] reported that low concentrations of microplastics did not have a significant impact on soil microbial diversity.

#### 4.2.5. Advances in Toxicological Research on MNPs and Human Health Risks

In recent years, research on MNPs has increasingly focused on ecological risk assessment and human exposure, encompassing Cluster #7 (“Risk Assessment”; 70 publications; S = 0.784; 2019) and Cluster #4 (“Human Exposure”; 80 publications; S = 0.833; 2020) (Figure 3e). In 2022, Heather AL et al. [127] were the first to detect plastic particles in human blood, revealing the widespread exposure of humans to submicron plastics. This finding has catalyzed further studies into their toxicological mechanisms and regulatory strategies, emphasizing the importance of MNPs as a public health challenge.

Based on the bibliometric co-citation network analysis (Q = 0.6729, S = 0.8410), research related to “humans” primarily focuses on the toxic effects of MNPs on human health. MNPs, ubiquitous pollutants that accumulate in the environment and food chain, have drawn widespread attention regarding human exposure and potential health risks. Since the first report of microplastics in the human intestine in 2018 [128], the presence of microplastics has also been detected in human lung [129], liver [130], spleen [130], and kidney tissues [130], and some studies have even identified microplastics in the placenta [127,131]. Microplastic particles have been detected in human feces [128], blood [127], lungs [132], and brain [133]. Among the three potential exposure routes—respiratory inhalation, gastrointestinal ingestion, and direct dermal contact—inhalation through the respiratory tract is likely the primary pathway for the entry of plastic particles into the human body [134]. Although the body can eliminate plastic particles via mucociliary clearance or pleural translocation [135], NPs with aerodynamic diameters less than 10 μm cannot be cleared by the body. These particles may penetrate deeply into the respiratory tract, even reaching the lung tissues, potentially leading to various respiratory diseases [135].

In the field of “Toxicology,” the bibliometric co-citation network analysis (Q = 0.6982, S = 0.8720) reveals a clear evolution in the stages of microplastic toxicity research. Early studies primarily focused on the macro-distribution and physical hazards of marine plastic pollution, discovering that direct threats to marine life mainly resulted from entanglement by large plastics and ingestion of microplastics [117], which shifted research priorities toward environmental pollutant behavior (Cluster #9, “Environmental Pollutants”; 23 publications; S = 0.953; 2008). Subsequent research delved into the toxicological mechanisms of microplastics, revealing that plastic additives are released during plastic degradation, particularly in the aging process caused by high temperatures and UV radiation, leading to the rapid release of large amounts of additives into the environment, which in turn harms ecosystems [136]. Researchers have detected concentrations of phthalate esters, a primary plastic additive, in freshwater fish, suggesting potential accumulation risks during gastrointestinal digestion [137]. Moreover, the longer microplastics persist in the environment, the greater the leaching of additives, leading to the accumulation of leachates with bioaccumulative effects in the food chain, posing threats to biological health [138,139] (Cluster #5, “Phthalates”; 43 publications; S = 0.941; 2011; Cluster #1, “Accumulation”; 58 publications; S = 0.825; 2015). Additionally, microplastics, as carriers of pollutants, enhance their toxicity. NPs, with their smaller particle size, exhibit greater biological hazard, affecting zooplankton [140] and algae [108], both directly and indirectly (Cluster #6, “Ecotoxicity”; 43 publications; S = 0.842; 2016).

Research on the toxicological effects of MNPs on organisms and humans has further diversified into three directions: First, the transfer of microplastics and their adsorbed pollutants through the food chain (Cluster #8, “Benzo[a]pyrene”; 30 papers; S = 0.859; 2016). Batel et al. [141] used fluorescence tracking to detect the adsorption of benzo[a]pyrene (BaP) on microplastic particles and its subsequent transfer to Artemia nauplii and further to zebrafish. Second, an in-depth exploration of the toxic mechanisms of microplastics (Cluster #4, “Proteomics”; 47 papers; S = 0.803; 2017). In aquatic organism research, Liu et al. [142] conducted a 21-day long-term exposure experiment on large Daphnia with polystyrene nanoparticles (PSNPs), showing that oxidative stress is the primary toxicological pathway. Nanoplastic exposure also significantly altered ribosomal structures, energy metabolism, and oxidative stress-related proteins in zebra mussels (Dreissena polymorpha) [143]. In mammalian models, co-exposure of polystyrene microplastics (PS) and the antibiotic ciprofloxacin (CIP) upregulated proteins related to bile secretion pathways in the liver of mice, leading to intrahepatic cholestasis. Long-term exposure suppressed energy metabolism pathways, increasing the risk of obesity [144]. Third, assessing the risk of human exposure to microplastics through consumption (Cluster #7, “Mussels”; 37 papers; S = 0.924; 2016). Various risk assessment methods have since been developed, including the hazard index method [145,146], pollution composite index method [147,148], and potential ecological risk index method [148,149], corresponding to Cluster #2 (“Pollution Load Index”; 56 papers; S = 0.860; 2018). By analyzing the microplastic concentrations in river [150], estuarine [146], and coastal [151] waters, these methods provide a systematic evaluation of environmental and ecological risks.

#### 4.2.6. Evolution of MNPs Research in Ecosystems and Emerging Trends

Based on bibliometric co-citation network analysis (Q = 0.6402, S = 0.8492), MNPs research under the theme of “ecosystem” demonstrates a clear evolutionary path and multidimensional focus. Research on MNPs in ecosystems is evolving from macro-distribution to micro-mechanisms, from single environmental media to cross-media interactions, and from physical hazards to chemical toxicology, reflecting a trend of multidimensional integration (Figure 3f). Early studies primarily focused on the fragmentation and degradation of plastic waste in marine environments, revealing the widespread presence and distribution characteristics of plastic debris. These studies laid the foundation for the basic understanding of the environmental behavior of MNPs (Cluster #1, “Plastic Debris”; 121 articles; S = 0.925; 2011). Marine microplastic pollution is a global issue. According to a report in Science, in 2010, 192 coastal countries and regions generated 275 million tons of plastic waste, of which approximately 8 million tons entered the oceans. By 2015, the figure had exceeded 9 million tons [152]. Under the combined effects of mechanical processes, biodegradation, photodegradation, and photooxidative degradation, plastic waste in the oceans is gradually broken down into smaller particles, persisting in the marine environment for centuries [153]. Microplastics either float in the seawater or settle on the seabed as part of the sediment, drawing widespread concern from researchers due to the potential risks they pose to marine ecosystems [154].

Subsequently, the research content gradually expanded and became more complex. On one hand, the focus shifted to the effects of MNPs on organisms, particularly their transmission and accumulation effects within the food chain. Toxicological experiments were conducted on typical marine organisms to assess ecological risks (Cluster #0, “Purple Mussel”; 148 articles; S = 0.776; 2016; Cluster #6, “Large Daphnia”; 97 articles; S = 0.781; 2016). Microplastics can block the feeding organs of marine animals. Studies have shown that a wide range of marine organisms, including zooplankton, benthic invertebrates, bivalves, fish, seabirds, and large marine mammals, can ingest microplastics. Once ingested, microplastics may cause mechanical damage, block food pathways, or create a false sense of satiety [155], leading to reduced feeding efficiency, energy depletion, injury, or even death [156]. On the other hand, the research scope has expanded to different environmental media and treatment processes, including water, soil, and wastewater treatment, emphasizing the migration, transformation, and removal efficiency of MNPs in the environment (Cluster #7, “Urban Streams”; 81 articles; S = 0.934; 2019; Cluster #2, “Estuaries”; 120 articles; S = 0.820; 2018; Cluster #4, “Wastewater Treatment Plants”; 108 articles; S = 0.844; 2017; Cluster #3, “Soil”; 118 articles; S = 0.902; 2018).

In recent years, polystyrene, as a typical plastic material, has been widely studied. Research has delved into its physicochemical properties and the adsorption mechanisms of pollutants on the microplastic surface, revealing the crucial impact of adsorption behavior on the migration and transformation of MNPs (Cluster #5, “Polystyrene”; 106 articles; S = 0.872; 2019; Cluster #8, “Adsorption”; 28 articles; S = 0.934; 2019). Furthermore, the exacerbating effects of global warming on the ecological risks of MNPs have gradually attracted attention, particularly in polar environments (Cluster #9, “Arctic Ocean”; 10 articles; S = 0.979; 2017). Polar sea ice, as the primary sink for historical anthropogenic particles, is continuously melting, releasing a large amount of trapped microplastics and exacerbating both polar and global marine pollution problems [51].

### 4.3. Future Research Trends in MNPs Pollution

**Figure 4:**
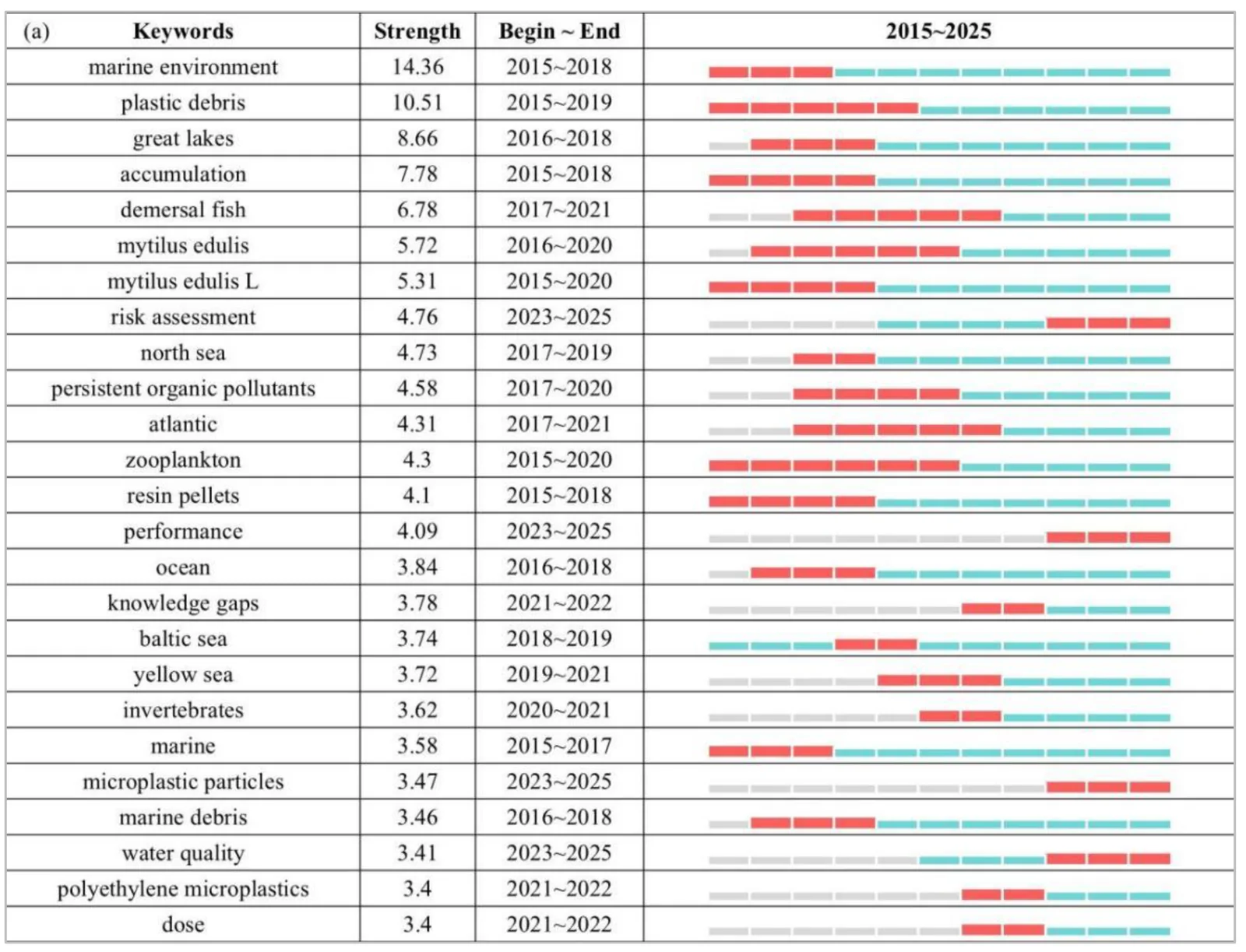

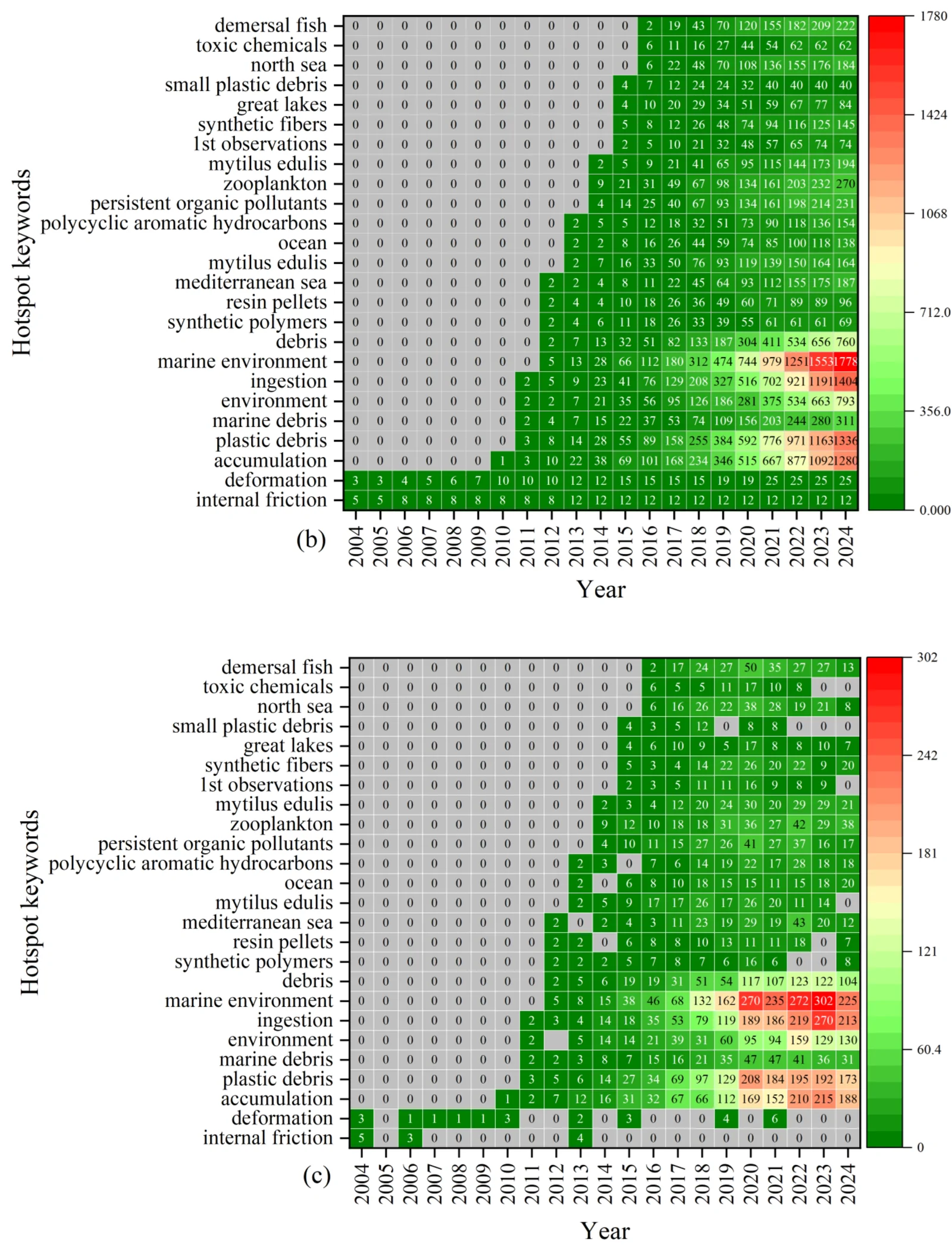
Prediction of microplastic research hotspot evolution based on keyword occurrence patterns. (**a**) Top 25 keywords with the strongest citation bursts by CiteSpace. The beginning of a blue line represents when an article is published. The beginning of a red mark is the beginning of a burst period and the end of the red mark is the end of the burst period. (**b**) Temporal variation in the cumulative occurrence frequency of hotspot keywords. (**c**) Temporal variation in the occurrence frequency of hotspot keywords.

Based on the temporal evolution and intensity analysis of the top 25 emerging keywords in the microplastic field from 1981 to 2025, future research hotspots reveal a multidimensional transformation trend (Figure 4a). Key issues such as “marine environment,” “cumulative effects,” “toxicity,” “ecological risks and environmental distribution,” and “plastic management” have emerged as central topics (Figure 4b,c). Future research will focus on advancing several key directions.

(1) Long-term threat of micro- and nanoplastics to marine ecosystems

As a major repository of global plastic flow, marine ecosystems have long been a core focus of research. Foundational studies have covered regions such as the North Atlantic, Indian Ocean, Arctic, and Pacific plastic gyres [157,158,159], establishing global plastic pollution baselines. Since 2013, the research network has rapidly expanded to include freshwater systems and terrestrial ecosystems, such as the Great Lakes, Rocky Mountain rivers, and agricultural soils, emphasizing the land-water-ocean plastic transfer chain. The detection of MNPs in extreme environments, such as Arctic Sea ice and deep-sea ecosystems, highlights that plastic pollution has become a global and cross-ecosystem challenge. The long-term impacts of MNPs on global ecosystems are expected to manifest first in marine ecosystems, making it crucial to closely monitor the destructive effects of marine microplastics.

(2) Comprehensive monitoring and dynamic analysis across environmental media

Although existing research predominantly focuses on marine ecosystems, the emergence of new keywords such as “atmospheric deposition,” “water quality,” and “environmental impact” since 2021 suggests that future microplastic studies will place greater emphasis on the interaction and transfer mechanisms between multiple media, including the atmosphere, water, and soil. This will push forward the establishment and standardization of an “integrated multi-layer monitoring system,” particularly in the development of nanoplastic monitoring technologies and the compatibility of data.

(3) Impact of microplastics on global biogeochemical cycles

Microplastic pollution affects Earth’s biogeochemical cycles, potentially influencing the functioning of ecosystems. Plastic pollution in aquatic and soil ecosystems can impact marine carbon storage, as well as nitrogen, phosphorus, and sulfur cycles [160,161,162]. In aquatic ecosystems, microplastics have been shown to inhibit phytoplankton photosynthesis (−45%) [163] and reduce marine primary productivity [164], thereby affecting energy flow and material cycles across the entire ecosystem and weakening its ability to maintain stability. Although numerous studies have demonstrated that widely distributed microplastics threaten ecosystem stability, the scattered evidence makes it difficult to predict direct consequences. Future research must delve deeper into the role and impact of microplastics on each process within ecosystems, providing more comprehensive evidence to better understand the ecological implications of microplastic disturbance. This will help establish a more scientifically grounded assessment system for the threat of microplastics to ecosystems, allowing for the prediction and prevention of potential ecological damage.

(4) Human toxicology of microplastics

The toxicity of microplastics to both plants and animals has been extensively studied and established. However, there remain several uncertainties and concerns regarding the potential toxicity of microplastics and their associated health risks to humans.

1. Microplastics in the environment vary significantly in terms of size, shape, and composition. What is the extent of their potential harm, and what are the underlying mechanisms of action?

2. Some research data lack biological significance or use unrealistic concentrations. For instance, the impact of microplastics on health can differ significantly depending on size. Studies have shown that particles larger than 1.5 micrometers may be too large to be absorbed by the body and are likely to be excreted via the digestive tract [165]. These particles cannot cross the air-blood barrier in the lungs [166], and any particle larger than 10 micrometers may also be unable to cross the gut-blood barrier [167]. Thus, the notion that particles larger than 10 micrometers may enter human tissues is difficult to accept.

3. The main scientific question is whether microplastics pose direct health risks to humans, what mechanisms underlie such potential toxicity, and how environmental exposure levels relate to these risks, given the current lack of conclusive evidence and systematic studies.

(5) Deepening the understanding of microscopic mechanisms and health risk assessment

The recent emergence of the keyword “genes” and its brief duration indicate that research is beginning to focus on the potential effects of MNPs at the molecular and cellular levels. Future research will increasingly concentrate on the bio-transport and toxicological mechanisms of NPs, as well as their potential risks to key health indicators such as the immune system, genetic material, and brain function. This will drive interdisciplinary collaborations for long-term low-dose accumulation effects and cross-generational exposure assessments. In terms of health risk assessment, the toxicity of microplastics shows strong particle size dependence and accumulation characteristics. At the cellular level, nanoscale plastics can penetrate the nuclear membrane and affect DNA. At the ecological level, medium and small-sized particles significantly inhibit the feeding behavior of zooplankton. Animal experiments suggest that high-dose exposure leads to inflammatory responses, but the exact exposure levels and long-term effects on humans have not yet been established. The accumulation of NPs in the human body and their potential neurotoxicity are beginning to emerge, and future research should focus on further elucidating the mechanisms and studying the cross-generational impacts.

(6) Standardization of nanoplastic detection techniques and data sharing

Currently, the broad range of MNPs particle sizes leads to scattered detection methods and inconsistencies in data. Future research will focus on overcoming challenges in the highly sensitive detection of NPs and promoting the development of international standards for sampling, preprocessing, and analysis. Strengthening the construction of open databases will facilitate efficient integration and application of cross-regional and interdisciplinary data. From a technical perspective, the wide particle size range of MNPs (from nanoscale to millimeter scale) leads to fragmentation in detection techniques, with data discrepancies between methods reaching as high as 400%. Despite the release of the first microplastic detection guidelines by ISO in 2020 [168], the integration of sampling and analysis protocols for NPs remains a significant challenge. Future technological innovations should focus on developing high-sensitivity nanoplastic detection methods, achieving standardization, and enhancing data compatibility for cross-regional comparisons.

(7) Understanding the ecological threats from microplastic interference

Existing evidence suggests that the widespread presence of microplastics is involved in nearly every process within ecosystems, influencing biodiversity through various pathways, such as environmental changes and species interactions. The impact extends across atmospheric, soil, freshwater, and marine ecosystems. Furthermore, microplastic interference can affect ecosystem productivity. Photosynthetic organisms are crucial to ecosystems as the primary producers of organic carbon [169]. However, existing studies indicate that microplastics generally suppress the chlorophyll a content of primary producers [170]. A modeled quantitative estimate reported in Reference [171] suggests that plastic exposure leads to a reduction in global photosynthesis by 7.05% to 12.12%, causing annual yield losses of 109.73 to 360.87 million tons in major global crops (rice, wheat, and maize). It should be noted that this estimate is derived from the published modeling work of Zhu et al. (2025) rather than outputs from our bibliometric analysis. The loss in net primary productivity (NPP) in marine and freshwater ecosystems ranges from 147.52 to 3415.11 million tons of carbon per year, which represents 0.31% to 7.24% of global NPP [171]. Therefore, the loss of ecosystem productivity caused by microplastic interference not only undermines ecosystem health but also presents challenges to human food security.

(8) Regional and ecosystem-specific risk assessment and management strategies

Between 2015 and 2020, there was a growing focus on specific ecosystems (such as the North Sea and the Great Lakes) and biological groups. This suggests that future research will focus on more refined analyses of the distribution and impacts of microplastic pollution across various geographic regions and ecosystems. By integrating environmental variables, differentiated risk assessment models and pollution control policies will be developed, thereby fostering regional governance and global policy coordination.

(9) Degradation of environmental plastics and development of alternative solutions

Conventional plastics have strong and stable long chains of hydrocarbons that degrade extremely slowly in the natural environment. Sunlight, heat, and bacteria are largely ineffective at breaking down their crystalline structure. Biodegradable plastics are considered a promising solution to plastic pollution. However, light- and oxygen-degradable plastics have been recognized as “pseudo-degradable” materials in many countries, and the degradation time and extent of fully biodegradable plastics (such as PLA and PBAT) remain controversial [84]. The United Nations Environment Programme (UNEP) published a 2019 report titled From Pollution to Solution: A Global Assessment of Marine Litter and Plastic Pollution, which stated that so-called “biodegradable plastics” are not a permanent solution and may cause environmental harm comparable to that of traditional plastics in terms of human health, biodiversity, and ecosystem functions [3].

In practice, the pathways and degradation conditions for biodegradable plastics are highly diverse and complex. The actual degradation of these materials is not well controlled, and the time required for complete degradation in natural environments varies depending on the product characteristics and environmental conditions. There is a lack of authoritative and universally applicable validation. Therefore, despite the widespread promotion of biodegradable plastics, environmental failure remains a significant issue. Future research in materials science will focus on developing truly environmentally friendly plastic alternatives, optimizing degradation performance, and assessing their ecological safety. This should be combined with environmental behavior studies to implement lifecycle pollution control.

(10) Improvement of a multi-layered management framework driven by policy

With the implementation of the United Nations Environment Programme (UNEP) Resolution to End Plastic Pollution and national plastic ban policies, future research should not only support policy development but also focus on scientifically evaluating and adjusting policy effectiveness. This will help form a management framework that integrates scientific, social, and economic dimensions, driving the coordination and upgrading of global plastic pollution governance systems.

## 5. Conclusions

This study employs bibliometric methods to systematically review and analyze the evolution, knowledge structure, and future trends of global research on MNPs from 1981 to early 2025. Based on a visualization of 12,851 publications indexed in the Web of Science Core Collection, we constructed a scientific knowledge map that reveals a clear trajectory of the field from its emergence to rapid development and gradual maturity. Research on MNPs has experienced exponential growth and increasingly globalized collaboration, with annual publications rising from 135 in 2016 to nearly 3000 in 2024, indicating that MNPs have become a central topic in environmental science. China, the United States, Germany, and the United Kingdom are the leading contributors to this domain. Co-citation network analysis identifies four significant research directions for the evolution of environmental distribution and migration, toxicological effects and health risks, source identification and environmental behavior, and the role of MNPs as vectors of co-contaminants, including the formation of unique microbial niches known as the “Plastisphere,” with their ecological functions and associated risks.

Research across different environmental media and subfields exhibits distinct evolutionary pathways. In soil systems, studies have shifted from source identification to investigations of physicochemical properties, microbial communities, and food chain effects. Freshwater research has focused on fluxes from terrestrial to marine systems and related ecological risks. Marine systems, as the original foundation of MNPs research, remain the most established subfield. The Plastisphere and human toxicology represent the most dynamic and emerging frontiers. Overall, this study delineates the developmental trajectory of MNPs research from distributional assessments to mechanistic exploration and further to risk evaluation and management strategies. The field is characterized by strong global relevance, interdisciplinarity, and complexity. Future efforts must overcome technological limitations in detection, advance understanding of human health impacts, and foster integration between natural and social sciences to provide a robust scientific foundation and policy support for addressing the global challenge of plastic pollution.

Nevertheless, several inherent caveats of the present bibliometric work should also be noted when interpreting these results. Despite the above-mentioned key findings of this bibliometric analysis, the present study still has several inherent limitations that should be acknowledged. First, citation accumulation is inherently time-dependent, meaning that many high-quality publications from recent years may not yet have achieved sufficient citation counts. This lag can bias the results and is partly attributable to the relatively slow uptake of emerging scientific frontiers. Consequently, co-citation networks may fail to fully capture the most recent research trends because newly published work is often under-cited.

Moreover, our subject-term-dependent search query TS = (“microplastics”) OR (“nanoplastics”) cannot comprehensively retrieve early publications from the 1970s–1980s that adopted alternative descriptive terms including plastic debris, microplastic, nanoplastic, microfibre, and polystyrene pellets. The formal term “microplastics” was not formally proposed until 2004, and pre-2004 studies seldom labeled microplastic-relevant research topics under these exact subject index terms in Web of Science Core Collection. Although these alternative wildcard-style search terms could potentially capture older literature, incorporating them into the TS-field query would retrieve substantial irrelevant records covering general solid-waste management, polymer-material engineering, and textile-chemistry research unrelated to environmental micro- and nanoplastic pollution, thereby introducing considerable dataset noise and contaminating the bibliometric analysis. This creates an inherent retrieval bias for older literature. Importantly, many landmark early works are still included in co-citation analyses through CiteSpace’s cited-reference expansion function; hence, these classic publications participate in co-citation clustering even if they are not directly matched by our subject-term search string. Readers should recognize this potential skewing effect on the original dataset.

Second, our analysis mainly considered first-author records exported from Web of Science, which may not fully reflect the broader scholarly influence of co-authors. Third, variations in author name formats and keyword expressions could introduce potential inconsistencies during clustering analysis, even though multiple rounds of manual checking have been performed. Furthermore, the automatic author-name extraction function of CiteSpace cannot completely disambiguate researchers with identical surname-initial combinations (e.g., Zhang Y, Liu Y), even though we have implemented manual curation for top-contributing author datasets as described in Supplementary Text S2. These caveats should be kept in mind when interpreting the results obtained from this bibliometric mapping.

## Data Availability

No new data were created or analyzed in this study. Data sharing is not applicable to this article.

## Supplementary Materials

The following supporting information can be downloaded at: https://www.mdpi.com/article/10.3390/toxics14090830/s1, Figure S1: Top 17 countries with the strongest beginning year of citation burst (2004–2025); Figure S2: Top 17 countries with the strongest strength of citation bursts (2004–2025); Figure S3: Top 25 institutions with the strongest strength of citation bursts (2004–2025); Figure S4: Top 25 institutions with the strongest beginning year of citation burst (2004–2025); Figure S5: Institutions and their cooperative relationships network related to MNPs; Figure S6: The network of cooperative relationships among countries and regions related to MNPs; Figure S7: The authors collaboration network around particular keywords; Figure S8: Top 25 authors with the strongest strength of citation bursts (2004–2025); Figure S9: Top 25 authors with the strongest beginning year of citation burst (2004–2025); Figure S10: Top 25 cited authors with the strongest strength of citation bursts (2004–2025); Figure S11: Top 25 cited authors with the strongest beginning year of citation burst (2004–2025); Figure S12: Top 25 cited journals with the strongest strength of citation bursts (2004–2025); Figure S13: Timeline visualization of co-occurring author keywords networks (2004–2025); Figure S14: Co-citation references network (2015–2025) and correspondent clustering analysis obtained with CiteSpace; Table S1: Top 10 most productive countries where papers on MNPs are published; Table S2: Top 10 most productive institutions where papers on MNPs are published; Table S3: Top 10 most published researchers on MPW; Table S4: Top 10 most productive journals where papers on MNPs are published; Table S5: The top ten disciplines covered by MNPs. References [172,173,174,175,176,177,178,179,180,181,182] cited in Supplementary Materials.
