# Supplementary material for: Global Trends in the Research Landscape and Development of Micro- and Nanoplastics
Source: Toxics. 2026 Sep 19;14(9):830. doi: 10.3390/toxics14090830 (PMC13611342; doi:10.3390/toxics14090830)
Supplement: Supplementary file 1 [file toxics-14-00830-s001.zip › toxics-4536064-supplementary.pdf]

Supplementary Materials

Global trends in the research landscape and development of micro- and nanoplastics

Yidan Luo[a, b#],Xinying Wu[b, c#], Wuhu Guo[b], Lele Ru[b], Ziyu Wei[b], Wending Huang[b], Wenhao Zhang[b], Evgeny Abakumov[d, e], Huoqing Xiao[a, b], Yuejin Chen[b], Jia Wang[f], Yanqi Liu[b], Hannan Ahmad Anjum[b], Sunlin Chi[b], Wei Liu[b]*, Xianchuan Xie[b]*

[a] Jiangxi Provincial Key Laboratory of Environmental Pollution Control, Jiangxi Academy of Eco-environmental Sciences and Planning, Nanchang, Jiangxi 330039, China

[b] Key Laboratory of Poyang Lake Environment and Resource Utilization, Engineering Research Center of Watershed Carbon Neutralization, Ministry of Education, School of Resource and Environment, Nanchang University, Nanchang 330031, China

[c] Anhui Nanxin Environmental Protection Technology Co, Ltd.10th Floor, Building 15, Life Science and Technology Park, No.8 Huangguan Road,High-tech Zone, Anqing City, Anhui Province 246005, China

[d] Department of Applied Ecology, Saint Petersburg State University, Saint Petersburg 199178, Russian Federation

[e] Analytical Research Laboratory Microplastics, Microplastics Research Center, A Yaroslav-the-Wise Novgorod State University, B. St. Petersburgskaya Str. 41, Veliky Novgorod 173003, Russia

[f] Jiangxi Key Laboratory of Watershed Soil and Water Conservation, Jiangxi Academy of Water Science and Engineering, Nanchang, Jiangxi 330029, China

# These authors contributed equally to this work.

* **Corresponding author.** Email: LiuW@ncu.edu.cn; chisunlin@163.com;

xchxie@ncu.edu.cn.

Supplementary information S1. Supplementary Text

S1. Methods

S1.1. Database selection

Web of Science (WoS) was founded in 1997 and is currently the largest comprehensive academic information resource database globally, covering the most disciplines. WoS's topic search is equivalent to a keyword search of some fields, including title, abstracts, author keywords, and keywords plus. Considering that the Web of Science Core Collection (WoSCC) is the one of the most suitable database collections for bibliometric studies (Mongeon and Paul-Hus, 2016), and that the full references and citations are mandatory for bibliometric analyses. To ensure the quality and accessibility of the data, we focus on the WOSCC database.

S1.2. Search strategy

The researchers will perform a wide-ranging search for related publications that consists of all field terms and phrases relevant to microplastic or nanoplastic (MNPs). For a more precise grasp of the topic, we have set a total of eight search themes. One is the overarching theme of micro - and nano - plastics, and the other seven are sub - themes that are relatively central within the realm of micro - and nano - plastics research, namely, ocean, water, soil, toxicity, microorganism, ecology, and human..We plan to search WOSCC from inception using the combination of keywords and MNPs terms.

The overarching theme search terms: TS = (Microplastic OR Nanoplastic).

The search formula for the ocean - related theme: TI=(Microplastic OR nanoplastic) AND TS = （Ocean OR Sea OR Marine OR Saltwater OR Blue water OR Open ocean OR Deep sea OR Pelagic zone OR Abyssal zone OR Continental shelf

OR Coastal waters OR Intertidal zone OR Estuarine waters OR Marine ecosystem OR Ocean currents OR Marine biodiversity OR Ocean Hydrosphere OR Marine pollution OR Aquatic environment OR Ocean basin OR Seafloor OR Maritime region OR Ocean).

The search formula for the water - related theme: TI = (Microplastic OR nanoplastic) AND TS = (Freshwater OR Fresh water OR Non-saline water OR Potable water OR Inland water OR Surface water OR Groundwater OR River water OR Lake water OR Stream water OR Wetland water OR Pond water OR Freshwater ecosystem OR Aquatic ecosystem OR Freshwater biodiversity OR Freshwater habitat OR Limnology OR Freshwater resource OR Water quality OR Hydrology OR Watershed OR Catchment area OR Rainwater OR River OR Lake OR Spring water OR Ice/Snowmelt OR Hydrosphere OR Contaminated water OR Stormwater OR Runoff OR Aquatic environment OR Aquifer OR Marsh OR Swamp OR Estuary OR Bay OR Inlet OR Depression OR Floodplain lake OR Stream OR Watercourse OR Waterway OR Brook OR Creek OR Tributary OR Channel OR Estuary OR Wetland OR Bayou OR Fjord OR Arroyo OR Drainage basin OR River delta OR Riverbank OR Riverbed OR Floodplain OR Main river OR Tributary river OR Delta river OR Floodplain river OR Seasonal river OR Perennial river OR Intermittent river/Ephemeral river OR Braided river OR Meandering river).

The search formula for the soil - related theme: TI = (Microplastic OR nanoplastic) AND TS = (Soil OR Ground OR Dirt OR Terrain OR Substrate OR Surface layer OR Pedosphere OR Soil profile OR Arable soil OR Farming soil OR Farmland OR Cultivated soil OR Loam OR Fertile soil OR Agricultural land OR Crop soil OR Silt soil OR Clay soil OR Urban soil OR Built soil OR Construction soil OR Industrial soil OR Polluted soil OR Contaminated soil OR Soil in urban areas OR Brownfield soil OR Terrestrial soil OR Land soil OR Dryland soil OR Forest soil OR Grassland soil OR Savanna soil OR Mountain soil OR Desert soil OR Wetland soil OR Peat soil OR Swamp soil OR Marsh soil OR Hydric soil OR Floodplain soil OR Riparian soil OR Mangrove soil OR Waterlogged soil OR Clay soil OR Sandy soil OR Loam soil OR Silt soil OR Peat soil OR Chalky soil OR Saline soil OR Alkaline

soil OR Acidic soil OR Healthy soil OR Soil fertility OR Soil structure OR Soil quality OR Soil composition OR Soil degradation OR Soil erosion OR Agricultural land OR Horticultural soil OR Garden soil OR Soil for construction OR Soil for landscaping OR Turf soil OR cropland)

The search formula for the toxicology - related theme: TI=(Microplastic OR nanoplastic) AND TS= (Toxicology OR Toxin studies OR Toxic effects OR Toxic substance research OR Toxicant analysis OR Hazard assessment OR Risk assessment OR Ecotoxicology OR Environmental toxicology OR Pollutant toxicity OR Chemical hazard assessment OR Contaminant toxicology OR Ecological risk assessment OR Biotoxicology OR Medical toxicology OR Biomedical toxicology OR Human toxicology OR Clinical toxicology OR Pathotoxicology OR Physiological toxicology OR Neurotoxicology OR Endocrine toxicology OR Reproductive toxicology OR Carcinogenicity studies OR Chemical toxicology OR Industrial toxicology OR Occupational toxicology OR Toxic chemical exposure OR Pesticide toxicology OR Heavy metal toxicity OR Pharmaceutical toxicology OR Nanotoxicology OR Food toxicology OR Nutritional toxicology OR Food safety assessment OR Foodborne toxin research OR Mycotoxin studies OR Additive toxicology OR Pharmacotoxicology OR Drug toxicity OR Pharmaceutical toxicology OR Adverse drug reactions OR Toxic pharmacology OR Xenobiotic toxicology OR Poison analysis OR Toxic substance detection OR Alcohol and drug screening)

The search formula for the ecology - related theme: TI = (Microplastic OR nanoplastic) AND TS = (Ecological OR Environmental OR Ecosystem OR Biological OR Biodiversity OR Eco-environmental OR Habitat-related OR Sustainability OR Soil ecology OR Soil ecosystem OR Edaphic ecology OR Soil biodiversity OR Rhizosphere ecology OR Pedosphere ecology OR Soil microbial ecology OR Terrestrial ecosystem OR Soil sustainability OR Soil fertility dynamics OR Aquatic ecology Freshwater ecology OR Water ecosystem OR Hydro-ecology OR River ecology OR Lake ecology OR Wetland ecology OR Aquatic biodiversity OR Lotic ecosystem OR Lentic ecosystem OR Hydrobiology OR Marine ecology OR Ocean ecology OR Sea ecology OR Marine ecosystem OR Pelagic ecosystem OR Coastal

ecology OR Deep-sea ecology Benthic ecology OR Estuarine ecology OR Marine biodiversity OR Ocean biogeochemistry OR Forest ecology OR Woodland ecosystem OR Canopy ecology OR Tropical ecology OR Temperate forest ecology OR Boreal forest ecology OR Forest biodiversity OR Silviculture ecology OR Grassland ecology OR Savanna ecosystem OR Steppe ecology OR Pasture ecology OR Rangeland ecology OR Prairie ecosystem OR Urban ecology OR Urban ecosystem OR Anthropogenic ecology OR Industrial ecology OR Metropolitan ecology OR Built environment ecology OR Desert ecology OR Polar ecology OR Tundra ecology OR High-altitude ecosystem OR Cave ecology OR Hydrothermal vent ecology)

The search formula for the microoganisms - related theme: TI = (Microplastic OR nanoplastic) AND TS = (Microorganisms OR Microbes OR Microbial life OR Microbial organisms OR Microbial flora OR Microbial community OR Bacteria OR Bacterium OR Prokaryotes OR Eubacteria OR Gram-positive bacteria OR Gram-negative bacteria OR Pathogenic bacteria OR Fungi OR Fungus OR Yeasts OR Molds OR Mushrooms OR Filamentous fungi OR Mycelium OR Viruses OR Viral particles OR Phages OR Viral genome OR Protozoa OR Protozoan OR Amoebae OR Ciliates OR Flagellates OR Parasitic protozoa OR Algae OR Phytoplankton OR Green algae OR Red algae OR Blue-green algae OR Seaweeds OR Unicellular organisms OR Single-celled life forms OR Microbial cells OR Cellular microorganisms OR Extremophiles OR Thermophiles OR Halophiles OR Acidophiles OR Alkaliphiles OR Psychrophiles)

The search formula for the human - related theme: TI = (Microplastic OR nanoplastic) AND TS = (Human being OR Person OR Individual OR Homo sapiens OR Mankind OR Humankind OR People OR Patient OR Human subject OR Human participant OR Clinical subject OR Human model)

We index in SCIEXPANDED, SSCI, A&HCI, ESCI, CCREXPANDED, IC, and search literature with the period of 1981-2025. After searching according to the above methods, 13690 records were available; we then screened the literature to be retrieved and finally included 11734 records in CiteSpace for quantitative analysis, including 10068 articles and 1419 reviews.

The data will be extracted from WOSCC as plain text file with full reference and citation for each document. We will inspect in detail all WOSCC highly cited references, the meeting abstracts, and the proceedings papers, for homogeneity of the retrieved dataset.

S1.3 Data collection

Two researchers independently analyzed the data to ensure the accuracy of the data and the repeatability of the research. Microsoft Excel 2021 was used to analyze and export the files of top-cited or productive authors, countries/regions, publications, journals, and institutions. In this review, CiteSpace 6.3.R1 (64-bit) was used to accomplish visualization to obtain insights into the research of MNPs and identify the research horizon and knowledge base of the field in large amounts of data.

S1.4. References to be included

There will be no limitation in the number of included references. Articles and reviews will be included, and each title of the available editorial materials and proceeding papers will be inspected for inclusion. In order to reflect the research trends, there will be no limitations on language, time, and populations.

S1.5. References to be excluded

Publications with irrelevant or out-of-scope topics will be excluded, such as: book chapters, monographs, conference papers or magazines. We will exclude publications categorized under corrections or retracted.

S2. Software and data analysis

Different software can be used to conduct scientometric analysis, that aims to produce systematic mapping, research trends, and influence networks. The different

software used automatically extract various data items, such as title, name of all authors, country, affiliation, author’s G-Index, number of citations, journals names, publication date, institutions names, keywords, keywords plus, funding’s, grants, major topic or subject area. In this study, we will use different software.

S2.1. CiteSpace (6. 3. R1)

CiteSpace is a Java application for visualizing and analyzing trends and patterns in scientific literature. It is designed as a tool for progressive knowledge domain visualization (Chen, 2006; Chen et al., 2010). It focuses on finding critical points in the development of a field or a domain, especially intellectual turning points and pivotal points.

We will use this software for production of both co-citation network and co-occurrence networks. In this paper, CiteSpace was adopted to generate visual knowledge maps, including countries, institutions, authors, journals, keyword clustering, and references citation bursts. Some knowledge map identification methods and parameters were involved in the results of the bibliometric analysis, which would be explained in paragraphs, respectively. Besides, a node indicated an item (country, institution, journal, author, or keyword), and links represented co-citation or co-occurrence between these nodes. Co-citation networks are based on co-citation, which is defined as a linkage between a pair of documents concurrently cited by a third document (Sabe et al., 2022; Stroup et al., 2000).

Co-citation networks extended from a single-slide equivalent to multiple-slice network analysis, i.e. a time series of networks in order to detect critical transitions over time more effectively. In CiteSpace, clusters are built on hybrid networks of co-cited articles and terms citing these articles.

Co-occurrence networks are the collective interconnection of terms based on their paired presence within a specified unit of text. We will use this software for production of both co-citation network and co-occurrence networks:

- Co-citation document networks

- Journal co-citation networks
- Author co-citation networks

Co-occurrence networks that are based on undirected graphs, directed graphs and hypergraphs:

- Co-occurrence authors keywords networks
- Co-authorship networks
- Co-authorship at the institutional level
- Co-authorship at the country level

S2.2 Manual author disambiguation

Raw author information exported from CiteSpace contains full given-name and surname combinations, which may bring inconsistencies including duplicated entries for the same author (name variants) and homonymous authors (different researchers sharing identical surname-initials combinations, e.g., Zhang Y, Liu Y).

The built-in automatic disambiguation function of CiteSpace relies merely on surname plus initials and cannot reliably separate distinct scholars with identical surname-initials. Therefore, manual curation was implemented for the dataset used to generate top-contributing-author statistics. We cross-checked author affiliations and corresponding publication titles to distinguish homonymous authors and filtered mixed records belonging to different researchers sharing identical surname-initials.

For bibliometric statistics and supplementary tables, only author surnames are presented; name initials are added for homonymous authors to avoid confusion. Full author given-name-surname information is preserved in the formal reference list, as required by academic citation standards.

S3 Evaluation index

Some indicators are used in this paper to evaluate countries, research institutions, researchers, journals and so on. These indicators can intuitively compare with their

influence. For example, the IFs of an academic journal is a measure reflecting the average number of citations to recent articles published in that journal, which is frequently used as a proxy for the relative importance of a journal within its field. Journals with higher impact factors are deemed to be more important than those with lower ones. H-index, also known as h-factor, is a new way to evaluate academic achievement. “H” stands for “high citations”, and a researcher's h-index means that he has at most h papers cited at least h times. The H-index can more accurately reflect a person's academic achievement. The higher the H-index of a person is, the greater the influence of his paper will be. For better or worse, academics use citation counts as one of several ways to assess the value of a researcher's output.

CiteSpace produces a variety of metrics of significance, with temporal metrics such as citation burstness, structural metrics such as betweenness centrality, modularity, and silhouette score as well as a combination of both, namely, the sigma metric. Betweenness centrality measures the number of times a node (e.g., one article, one author) lies on the shortest path between other nodes based on Freeman’s betweenness centrality metric (Freeman, 1977). Nodes with high betweenness centrality generally connect different clusters and are considered key hubs. Burstness measures the rate of change. The burstness of the frequency of an entity over time indicates a specific duration when an abrupt change in the frequency takes place, thus identifying emergent terms (Kleinberg, 2003). CiteSpace also combines betweenness centrality as a structural property with citation burstness as a temporal property using a metric called sigma. Sigma is computed as $(\text{centrality} + 1)^{\text{burstness}}$ (Chen et al., 2010) with higher values indicating works with higher influential potential. The modularity (the Q score) of a network measures the extent to which a network can be divided into modules or clusters, and the silhouette (the S score) is a method of interpretation and validation of consistency within clusters of data (Shibata et al., 2008). The Q metric ranges from 0 to +1, and the S metric ranges from −1 to +1. For both metrics, a score close to +1 represents the best clustering model. When the Q value is greater than 0.3, the cluster structure is considered significant, and higher values may indicate a well-structured network; when the silhouette coefficients exceed 0.3, 0.5, or 0.7, the

network is considered homogenous, reasonable, or highly credible, respectively. A silhouette score of 1 may, however, indicate that the corresponding cluster is relatively isolated. The cluster labels are generated from noun phrases of the keyword lists of articles cited in each cluster using the likelihood ratio test ($p < 0.001$). Each cluster was closely inspected, and automatic labels were re-labelled according to authors' expertise, if needed. The impact factors of the included journals were retrieved from the 2021 Journal Citation Reports (data were extracted from WOSCC in plain text files).

The g-index, an author-level metric based on the distribution of citations received that alleviates bias from highly cited papers as seen with the H-index, was used for all calculations (Egghe, 2007). Of importance, the inflated values of the g-Index help to give credit to lowly-cited or non-cited papers while giving credit for highly-cited papers, making g-index most relevant for co-cited analysis.

S3.1. Network analysis metrics and data analysis

Different network metrics will be used in our analysis, such as betweenness centrality, burstness analysis, sigma scores, and modularity and silhouette scores.

①**Betweenness centrality:** The algorithm calculates unweighted shortest paths between all pairs of nodes in a graph. Each node receives a score, based on the number of shortest paths that pass through the node (Freeman, 1977).

②**Burstness analysis:** Citation burst is a detection of a burst event (Kleinberg, 2003). A citation burst provides evidence that a particular publication is associated with a surge of citations. Furthermore, if a cluster contains numerous nodes with strong citation bursts, then the cluster as a whole captures an active area of research, or an emerging trend.

③**Modularity scores:** Modularity is a measure of the structure of a graph, measuring the density of connections within a module. Graphs with a high modularity score will have many connections within a module but only few pointing outwards to other modules. Its value ranges from 0 to 1 (Newman, 2006).

④**Silhouette scores:** Silhouette score is a metric used to calculate the goodness of a clustering technique (Rousseeuw, 1987). Its value ranges from -1 to 1.

1: clusters are isolated from each other.

0: clusters are indifferent, or we can say that the distance between clusters is not significant.

-1: clusters are assigned in the wrong way.

⑤**Sigma scores:** This indicator measures the combined strength of structural and temporal properties of a node, namely, its betweenness centrality and citation burst (Chen et al., 2012).

S3.2. Additional data extraction

We will extract top unit of measure for a specific time period:

- The top 10 cited units of measure (e.g. top 10 cited journals)
- The top 10 most productive units of measure (e.g. top 10 most cited reference)
- Journal's impact factors
- Journal's year of first appearance
- Author full names

S4. Sensitivity analyses

When adequate, we will reduce the time period to the last 5 years (2016-2021), and/or to the last year (2021) in order to further explore latest research trends of modifications of unit of measure. Specific unit of measure will be extracted with different software in order to inspect different results (e.g. keywords). If possible, we will conduct structural variation analysis using centrality and modularity divergence to monitor and discern the potential of newly published papers in context.

S5. Data availability

The extracted database will be seared upon reasonable request.

Supplementary information S2. Supplementary Tables

Researchers retrieved literature from the Science Citation Index Expanded (SCI - EXPANDED), Social Sciences Citation Index (SSCI), and Conference Proceedings Citation Index - Science (CPCI - S) within the Web Science Core Collection using the search formula for the overarching topic. The retrieval time span was from January 1, 1981, to December 31, 2024. Data such as the total number of publications and the total number of citations were exported and presented in tables.

Table S1. Top 10 most productive countries where papers on MNPs are published.

Number	Country	TA	P (%)	H-index	TC	CPA	Centrality
1	PR. China	3,838	30.98	213	214,520	55.89	0.04
2	USA	1,353	10.92	159	101,725	75.18	0.11
3	Germany	1,020	8.23	148	93,203	91.38	0.09
4	India	787	6.35	89	30,773	39.10	0.07
5	England	731	5.90	151	103,367	141.40	0.15
6	Italy	706	5.70	97	38,855	55.04	0.08
7	South Korea	593	4.79	85	30,517	51.46	0.04
8	Spain	563	4.55	81	29,620	52.61	0.17
9	Austrulia	515	4.16	112	52,199	101.36	0.06
10	Canada	514	4.15	96	42,219	82.14	0.04

*Note: TA: total articles; TC: total citation count; CPA: average number of citations per article; P: proportion.

Table S2. Top 10 most productive institutions where papers on MNPs are published.

Number	Institution	TA	P (%)	H-index	TC	CPA	Country	Centrality
1	CAS	639	5.16	113	45,681	71.49	PR. China	0.07
2	UCAS	280	2.26	75	22,565	80.59	PR. China	-
3	CNRS	274	2.21	72	23,183	84.61	France	-
4	HGF	224	1.81	71	25,540	114.02	Germany	-
5	ECNU	192	1.55	83	26,564	138.35	PR. China	-
6	CNR	167	1.35	51	10,389	62.21	Italy	0.03
7	WUR	164	1.32	76	27,988	170.66	Holand	0.02
8	MNR	159	1.28	50	7,936	49.91	PR. China	0.05

9	RAS	142	1.15	26	3,439	24.22	Russia	-
10	CSIC	127	1.03	37	6,855	53.98	Spain	-

*Note: CAS: Chinese Academy of Sciences; UCAS; University of Chinese Academy of Sciences CAS; CNRS: Centre National de la Recherche Scientifique CNRS; HGF: Helmholtz Association; ECNU: East China Normal University; CNR: Consiglio Nazionale delle Ricerche CNR; WUR: Wageningen University Research; MNR: Ministry of Natural Resources of the People's Republic of China; RAS: Russian Academy of Sciences; CSIC: Consejo Superior de Investigaciones Científicas CSIC..

Table S3. Top 10 most published researchers on MPW.

Number	Author	TA	P (%)	H-index	TC	CPA	Institution	Country
1	Koelmans	74	0.60	49	17,659	238.64	Wageningen University & Research	Netherlands
2	Rochman	72	0.58	41	7,360	102.22	Univ Toronto	Canada
3	Shi	69	0.56	53	15,405	223.26	East China Normal University	PR. China
4	Thompson	67	0.54	53	36,952	551.52	University of Plymouth	England
5	Rillig	55	0.44	31	10,486	190.65	Free University Berlin	Germany
6	Laforsch	54	0.44	28	5,658	104.78	University of Bayreuth	Germany
7	Li	48	0.39	29	7,762	161.71	East China Normal Univ	PR. China
8	Lusher	43	0.35	30	9,211	214.21	NIVA	Norway
9	Wang, J	42	0.34	29	5,760	137.14	South China Agricultural University	PR. China
10	Wang, D	42	0.34	27	1,590	37.86	Southeast University	PR. China

*Note: NIVA: Norwegian Institute for Water Research

Table S4. Top 10 most productive journals where papers on MNPs are published.

Number	Journal	TA	P (%)	H-index	TC	CPA	IF
1	SCIENCE OF THE TOTAL ENVIRONMENT	1,489	12.02	162	110,908	74.48	8
2	MARINE POLLUTION BULLETIN	1,072	8.65	138	84,113	78.46	4.9
3	ENVIRONMENTAL	919	7.42	160	95,567	103.99	7.3

	POLLUTION						
4	JOURNAL OF HAZARDOUS MATERIALS	666	5.38	95	34,352	51.58	11.3
5	ENVIRONMENTAL SCIENCE TECHNOLOGY	361	2.91	125	73,968	204.90	11.3
6	CHEMOSPHERE	347	2.80	95	29,617	85.35	8.1
7	WATER RESEARCH	241	1.95	88	32,811	136.15	12.4
8	ECOTOXICOLOGY AND ENVIRONMENTAL SAFETY	167	1.35	45	7,405	44.34	6.1
9	WATER	150	1.21	33	3,317	22.11	3
10	FRONTIERS IN MARINE SCIENCE	145	1.17	36	4,387	30.26	3

Table S5. The top ten disciplines covered by MNPs

Number	Discipline classification	TA	P (%)	H-index	TC	CPA
1	Environmental Sciences	8,256	66.65	339	605,018	73.28
2	Engineering Environmental	1,924	15.53	194	167,061	86.83
3	Marine Freshwater Biology	1,739	14.04	153	108876	62.61
4	Water Resources	855	6.90	102	47,066	55.05
5	Toxicology	847	6.84	102	41,517	49.02
6	Materials Science Multidisciplina	502	4.05	56	13,714	27.32
7	Chemistry Multidisciplinary	468	3.78	63	18,154	38.79
8	Multidisciplinary Sciences	441	3.56	93	40,327	91.44
9	Chemistry Analytical	401	3.24	79	24,097	60.09
10	Engineering Chemical	400	3.23	53	10,006	25.02

Supplementary information S3. Supplementary Figures

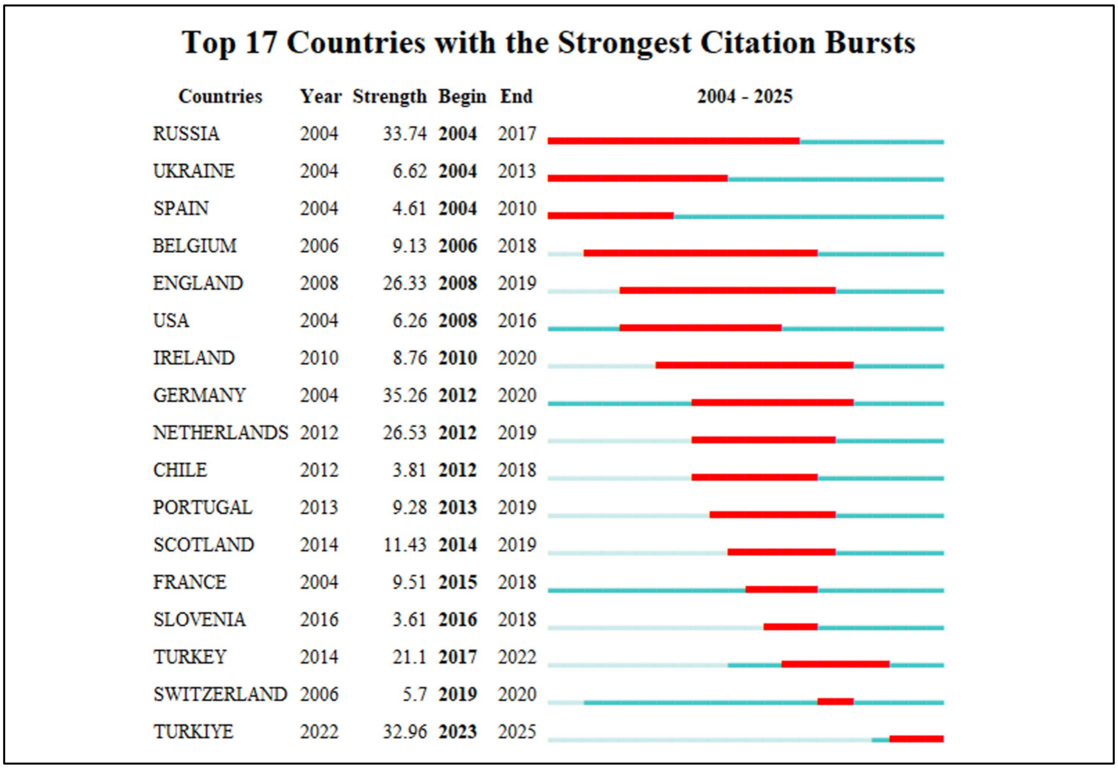

Fig. S1 Top 17 countries with the strongest beginning year of citation burst (2004-2025).

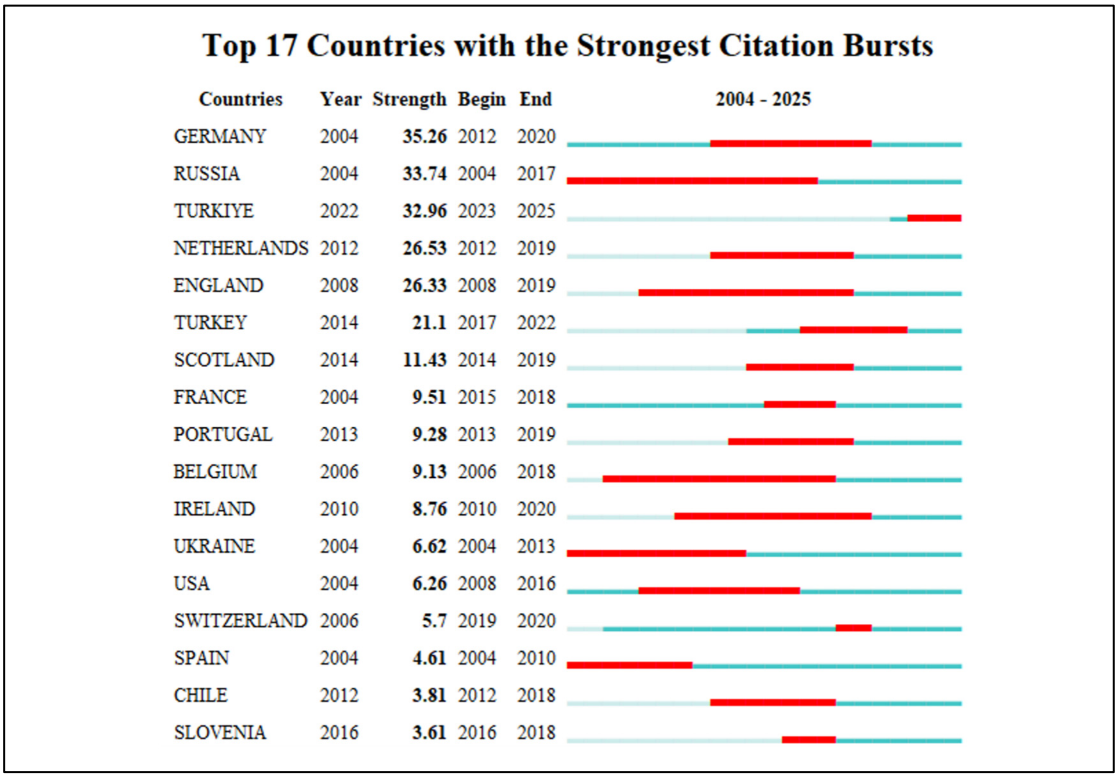

Fig. S2 Top 17 countries with the strongest strength of citation bursts (2004-2025).

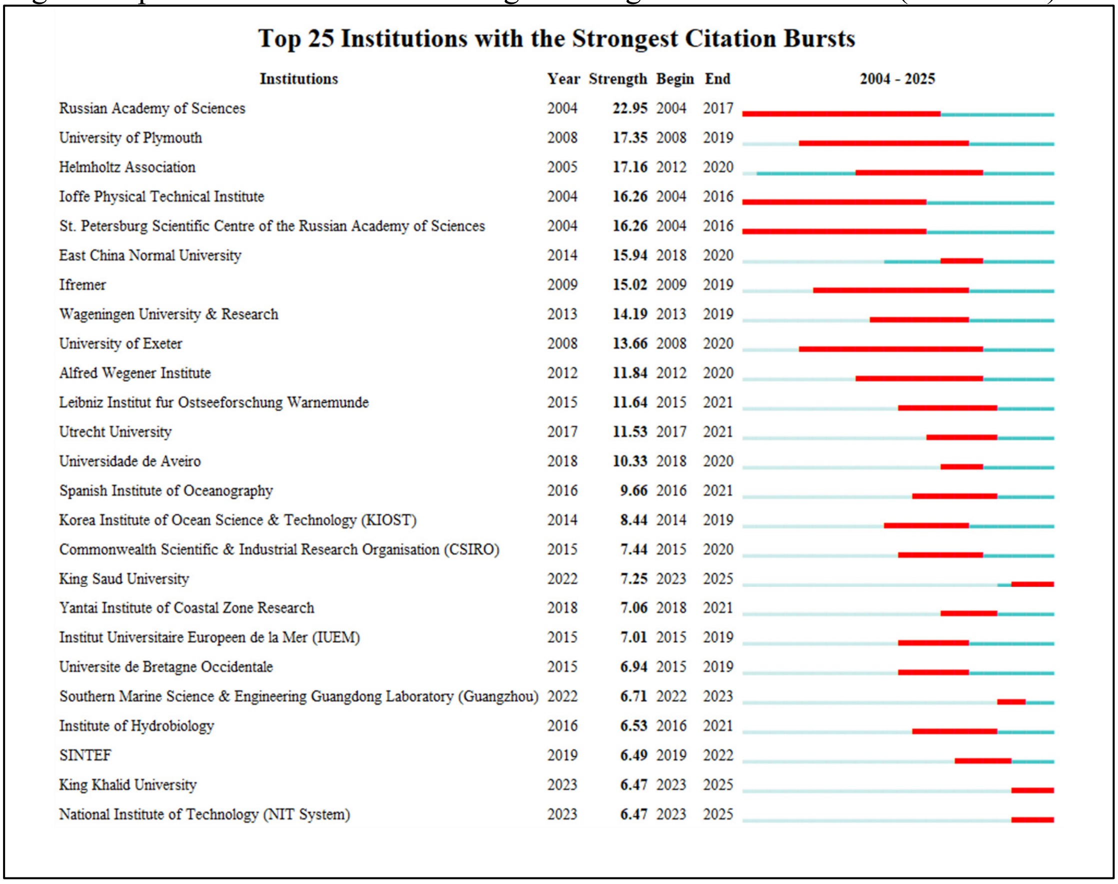

Top 25 Institutions with the Strongest Citation Bursts

Institutions	Year	Strength	Begin	End	2004 - 2025
Russian Academy of Sciences	2004	**22.95**	2004	2017	
University of Plymouth	2008	**17.35**	2008	2019	
Helmholtz Association	2005	**17.16**	2012	2020	
Ioffe Physical Technical Institute	2004	**16.26**	2004	2016	
St. Petersburg Scientific Centre of the Russian Academy of Sciences	2004	**16.26**	2004	2016	
East China Normal University	2014	**15.94**	2018	2020	
Ifremer	2009	**15.02**	2009	2019	
Wageningen University & Research	2013	**14.19**	2013	2019	
University of Exeter	2008	**13.66**	2008	2020	
Alfred Wegener Institute	2012	**11.84**	2012	2020	
Leibniz Institut fur Ostseeforschung Warnemunde	2015	**11.64**	2015	2021	
Utrecht University	2017	**11.53**	2017	2021	
Universidade de Aveiro	2018	**10.33**	2018	2020	
Spanish Institute of Oceanography	2016	**9.66**	2016	2021	
Korea Institute of Ocean Science & Technology (KIOST)	2014	**8.44**	2014	2019	
Commonwealth Scientific & Industrial Research Organisation (CSIRO)	2015	**7.44**	2015	2020	
King Saud University	2022	**7.25**	2023	2025	
Yantai Institute of Coastal Zone Research	2018	**7.06**	2018	2021	
Institut Universitaire Europeen de la Mer (IUEM)	2015	**7.01**	2015	2019	
Universite de Bretagne Occidentale	2015	**6.94**	2015	2019	
Southern Marine Science & Engineering Guangdong Laboratory (Guangzhou)	2022	**6.71**	2022	2023	
Institute of Hydrobiology	2016	**6.53**	2016	2021	
SINTEF	2019	**6.49**	2019	2022	
King Khalid University	2023	**6.47**	2023	2025	
National Institute of Technology (NIT System)	2023	**6.47**	2023	2025	

Fig. S3 Top 25 institutions with the strongest strength of citation bursts (2004-2025).

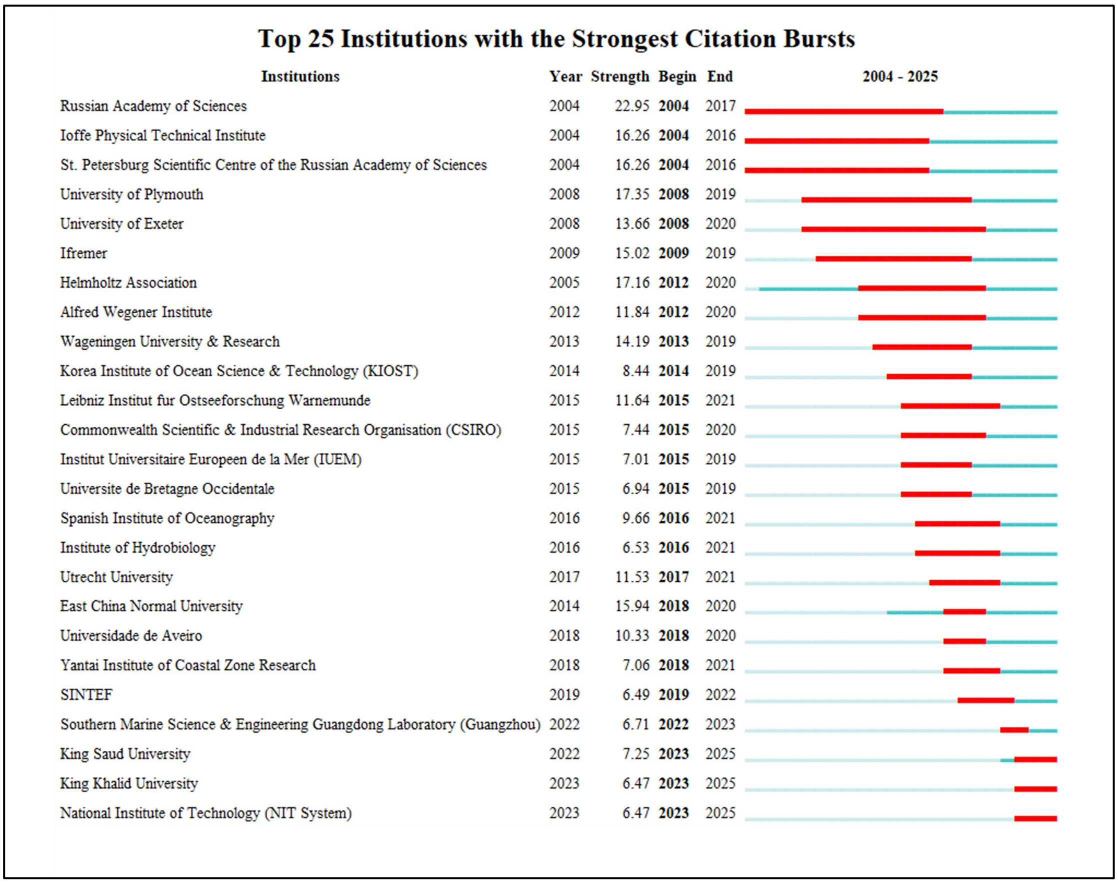

Top 25 Institutions with the Strongest Citation Bursts

Institutions	Year	Strength	Begin	End	2004 - 2025
Russian Academy of Sciences	2004	22.95	**2004**	2017	
Ioffe Physical Technical Institute	2004	16.26	**2004**	2016	
St. Petersburg Scientific Centre of the Russian Academy of Sciences	2004	16.26	**2004**	2016	
University of Plymouth	2008	17.35	**2008**	2019	
University of Exeter	2008	13.66	**2008**	2020	
Ifremer	2009	15.02	**2009**	2019	
Helmholtz Association	2005	17.16	**2012**	2020	
Alfred Wegener Institute	2012	11.84	**2012**	2020	
Wageningen University & Research	2013	14.19	**2013**	2019	
Korea Institute of Ocean Science & Technology (KIOST)	2014	8.44	**2014**	2019	
Leibniz Institut fur Ostseeforschung Warnemunde	2015	11.64	**2015**	2021	
Commonwealth Scientific & Industrial Research Organisation (CSIRO)	2015	7.44	**2015**	2020	
Institut Universitaire Europeen de la Mer (IUEM)	2015	7.01	**2015**	2019	
Universite de Bretagne Occidentale	2015	6.94	**2015**	2019	
Spanish Institute of Oceanography	2016	9.66	**2016**	2021	
Institute of Hydrobiology	2016	6.53	**2016**	2021	
Utrecht University	2017	11.53	**2017**	2021	
East China Normal University	2014	15.94	**2018**	2020	
Universidade de Aveiro	2018	10.33	**2018**	2020	
Yantai Institute of Coastal Zone Research	2018	7.06	**2018**	2021	
SINTEF	2019	6.49	**2019**	2022	
Southern Marine Science & Engineering Guangdong Laboratory (Guangzhou)	2022	6.71	**2022**	2023	
King Saud University	2022	7.25	**2023**	2025	
King Khalid University	2023	6.47	**2023**	2025	
National Institute of Technology (NIT System)	2023	6.47	**2023**	2025	

Fig. S4 Top 25 institutions with the strongest beginning year of citation burst (2004-2025).

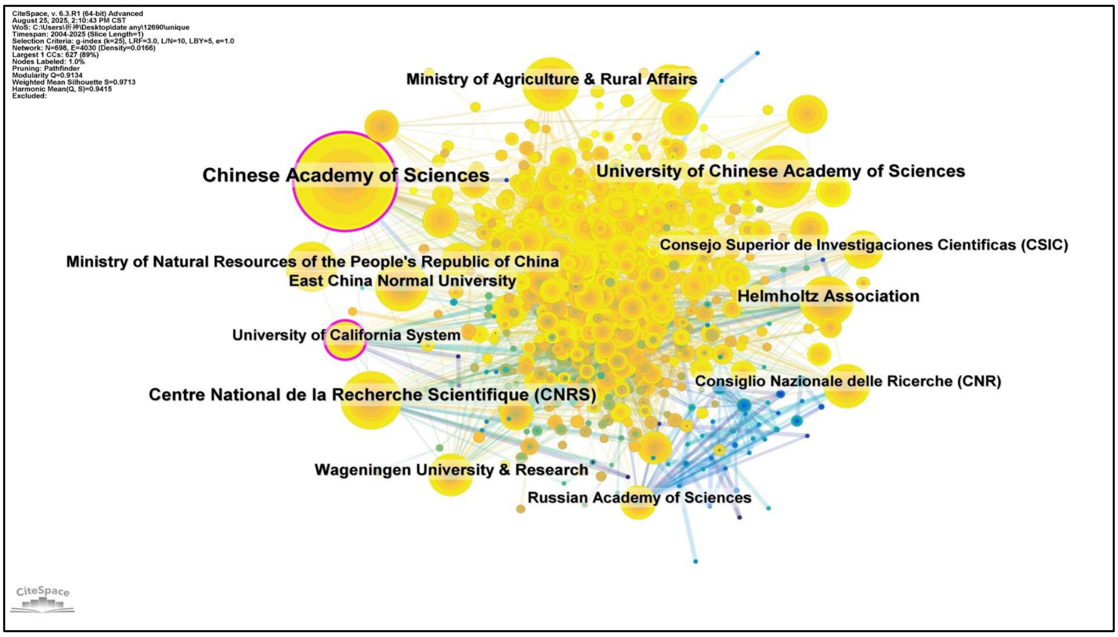

Fig. S5 Institutions and their cooperative relationships network related to MNPs

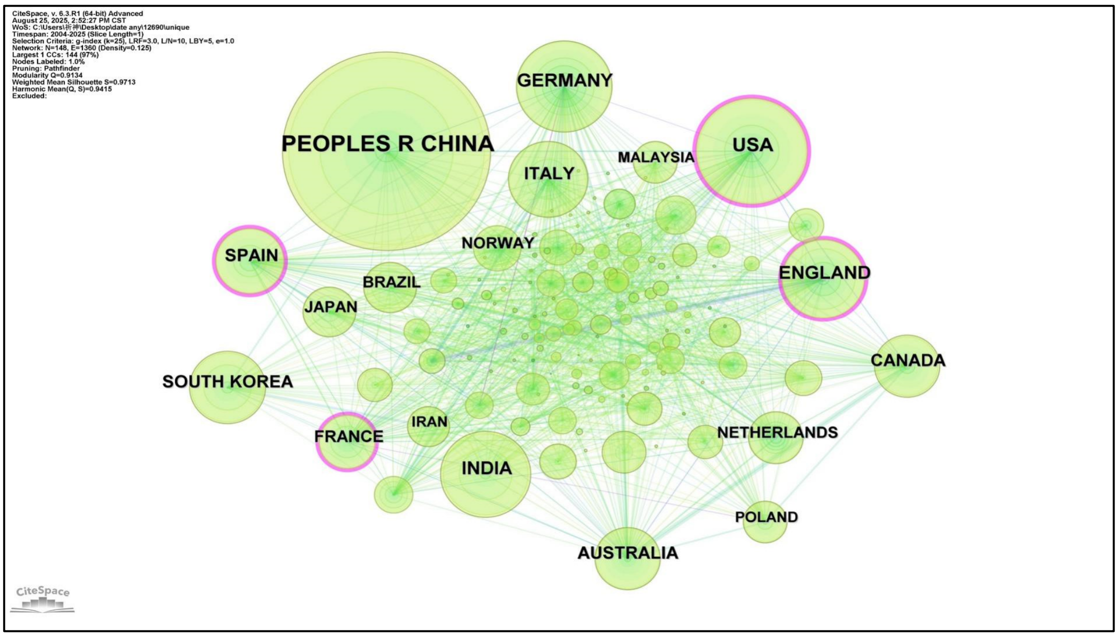

Fig. S6 The network of cooperative relationships among countries and regions related to MNPs

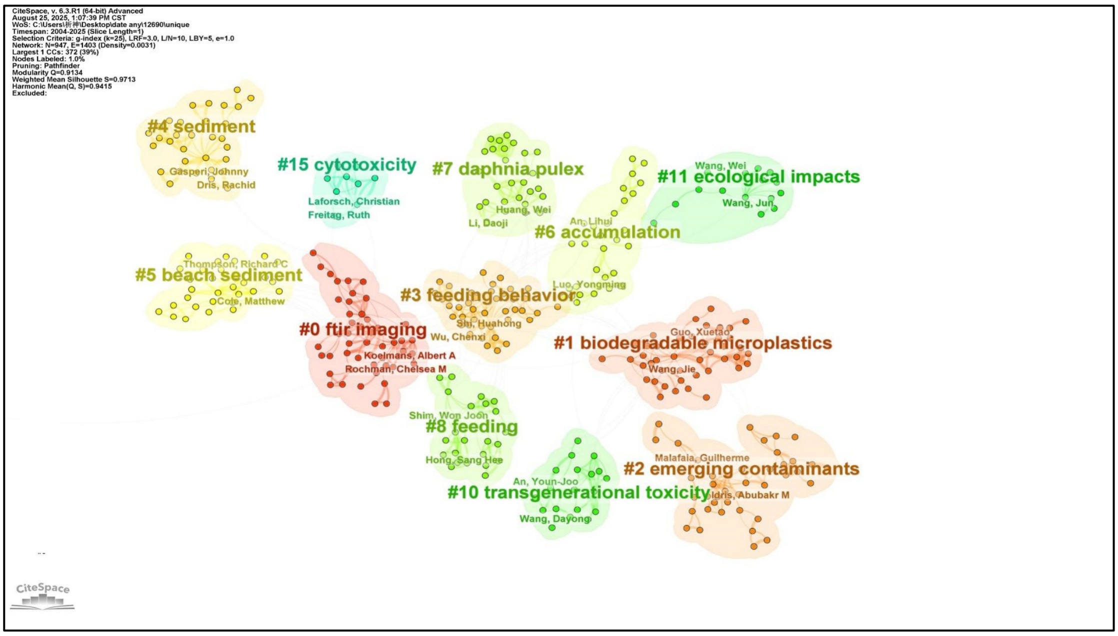

Fig. S7 The authors collaboration network around particular keywords.

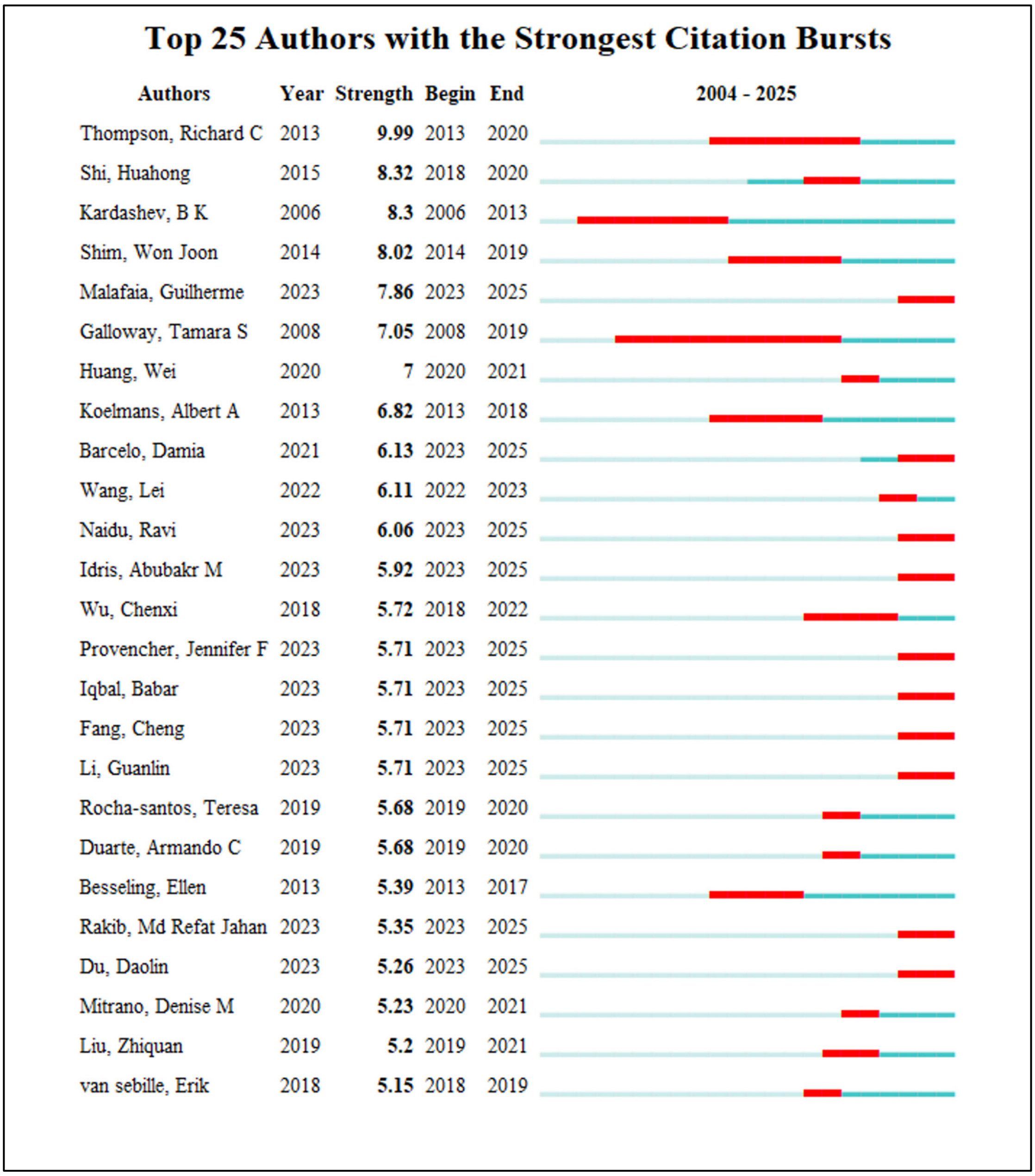

Fig. S8 Top 25 authors with the strongest strength of citation bursts (2004-2025).

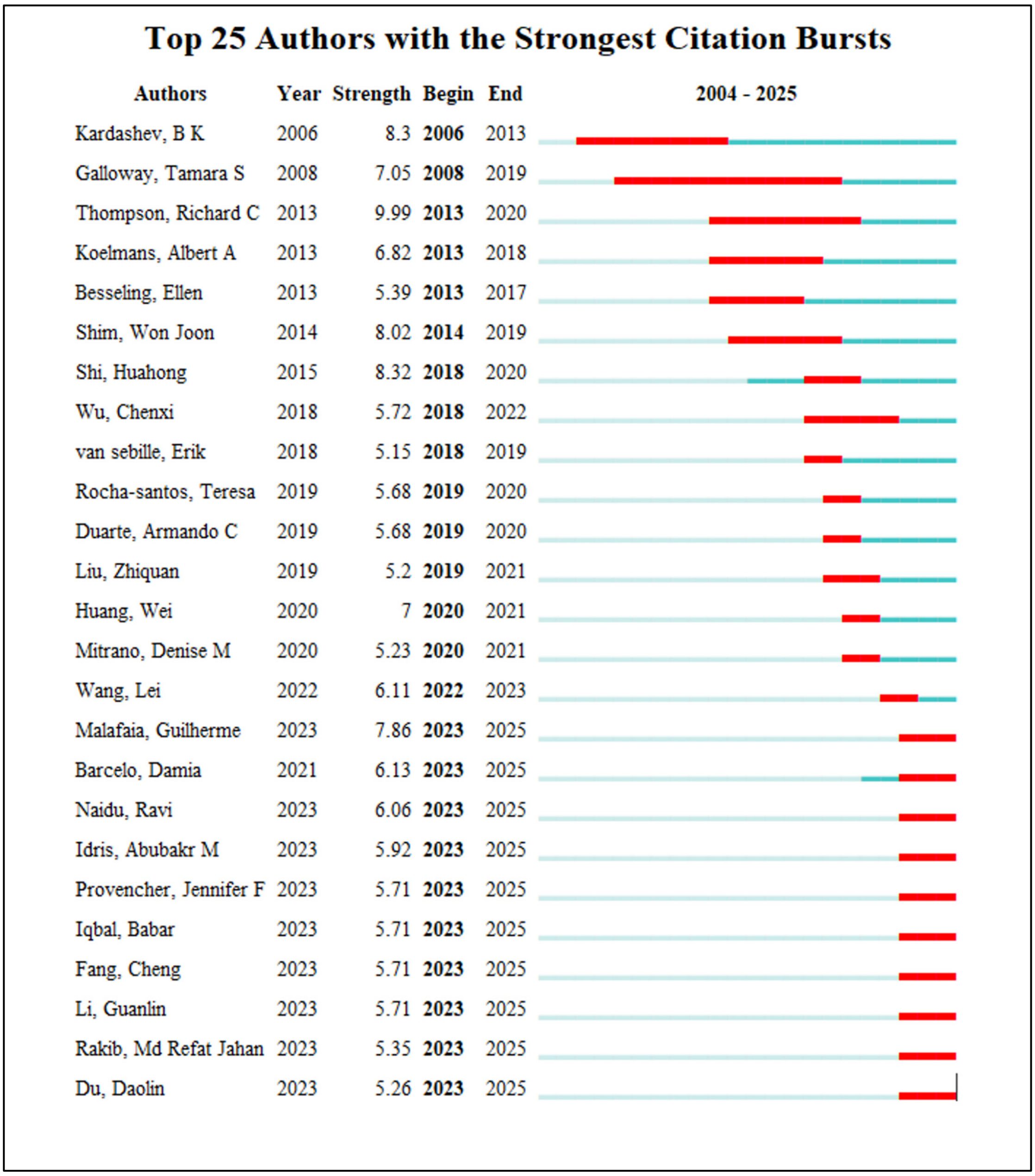

Fig. S9 Top 25 authors with the strongest beginning year of citation burst (2004-2025).

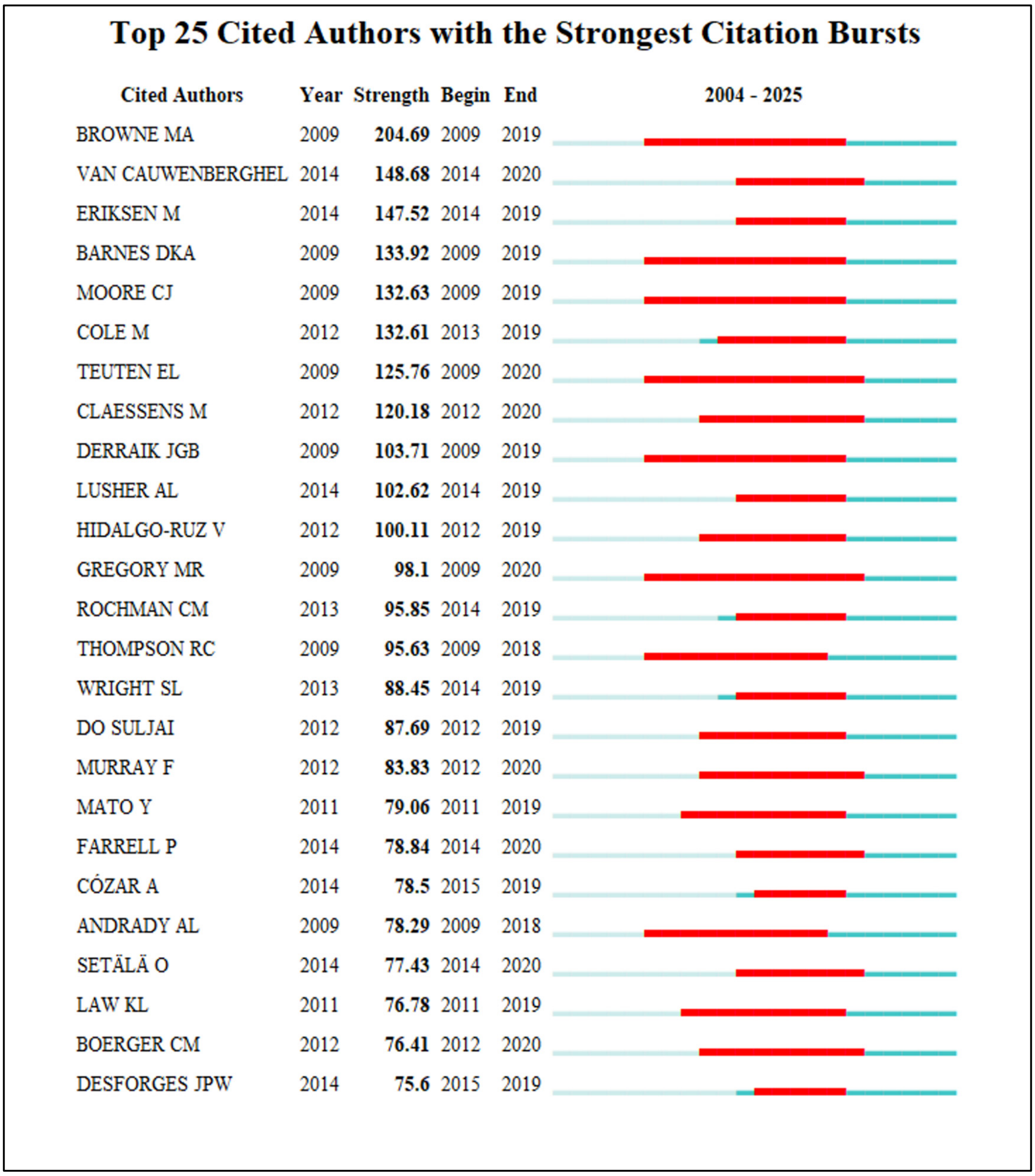

Top 25 Cited Authors with the Strongest Citation Bursts

Cited Authors	Year	Strength	Begin	End	2004 - 2025
BROWNE MA	2009	**204.69**	2009	2019	
VAN CAUWENBERGHEL	2014	**148.68**	2014	2020	
ERIKSEN M	2014	**147.52**	2014	2019	
BARNES DKA	2009	**133.92**	2009	2019	
MOORE CJ	2009	**132.63**	2009	2019	
COLE M	2012	**132.61**	2013	2019	
TEUTEN EL	2009	**125.76**	2009	2020	
CLAESSENS M	2012	**120.18**	2012	2020	
DERRAIK JGB	2009	**103.71**	2009	2019	
LUSHER AL	2014	**102.62**	2014	2019	
HIDALGO-RUZ V	2012	**100.11**	2012	2019	
GREGORY MR	2009	**98.1**	2009	2020	
ROCHMAN CM	2013	**95.85**	2014	2019	
THOMPSON RC	2009	**95.63**	2009	2018	
WRIGHT SL	2013	**88.45**	2014	2019	
DO SULJAI	2012	**87.69**	2012	2019	
MURRAY F	2012	**83.83**	2012	2020	
MATO Y	2011	**79.06**	2011	2019	
FARRELL P	2014	**78.84**	2014	2020	
CÓZAR A	2014	**78.5**	2015	2019	
ANDRADY AL	2009	**78.29**	2009	2018	
SETÄLÄ O	2014	**77.43**	2014	2020	
LAW KL	2011	**76.78**	2011	2019	
BOERGER CM	2012	**76.41**	2012	2020	
DESFORGES JPW	2014	**75.6**	2015	2019	

Fig. S10 Top 25 cited authors with the strongest strength of citation bursts (2004-2025).

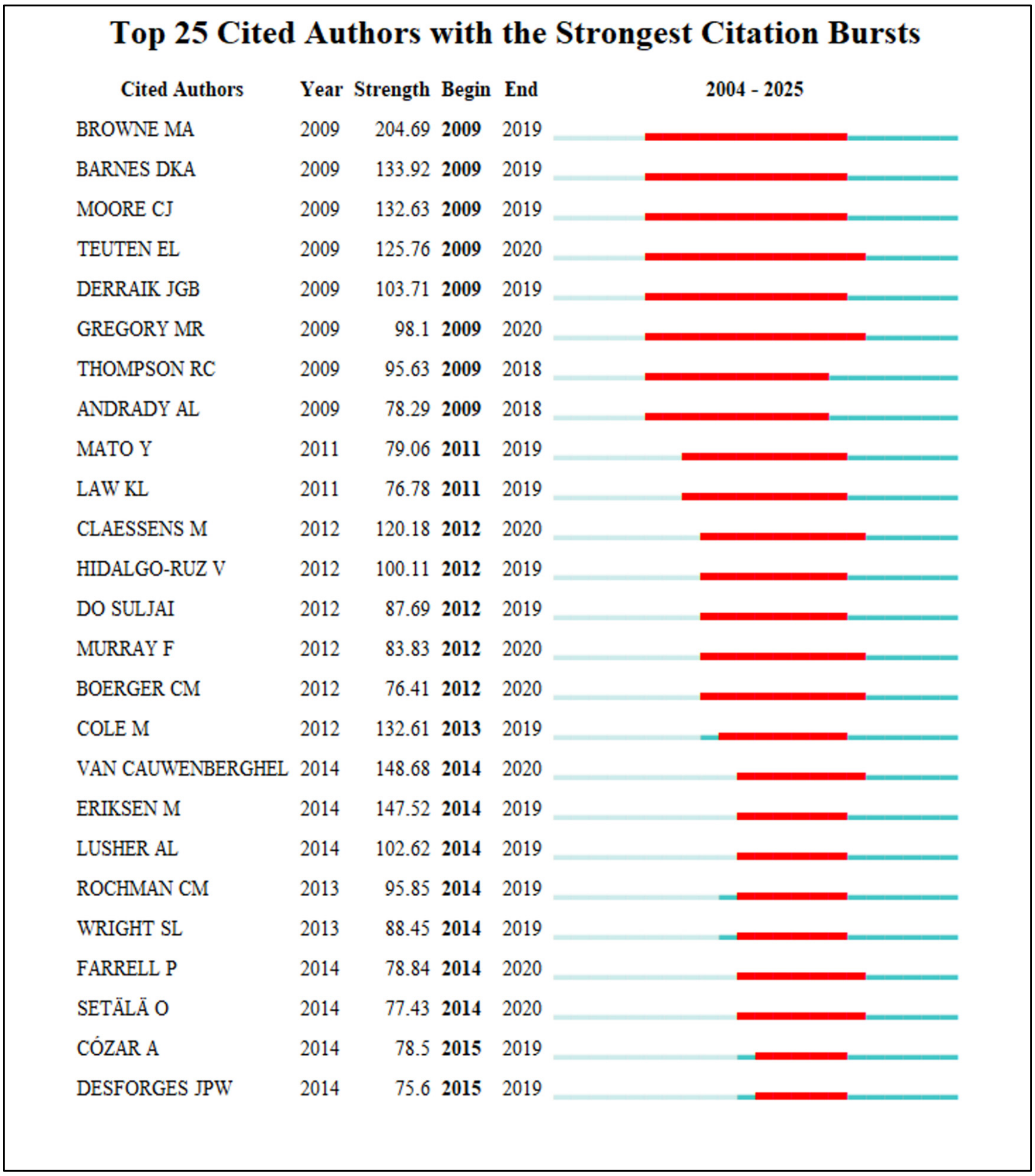

Fig. S11 Top 25 cited authors with the strongest beginning year of citation burst (2004-2025).

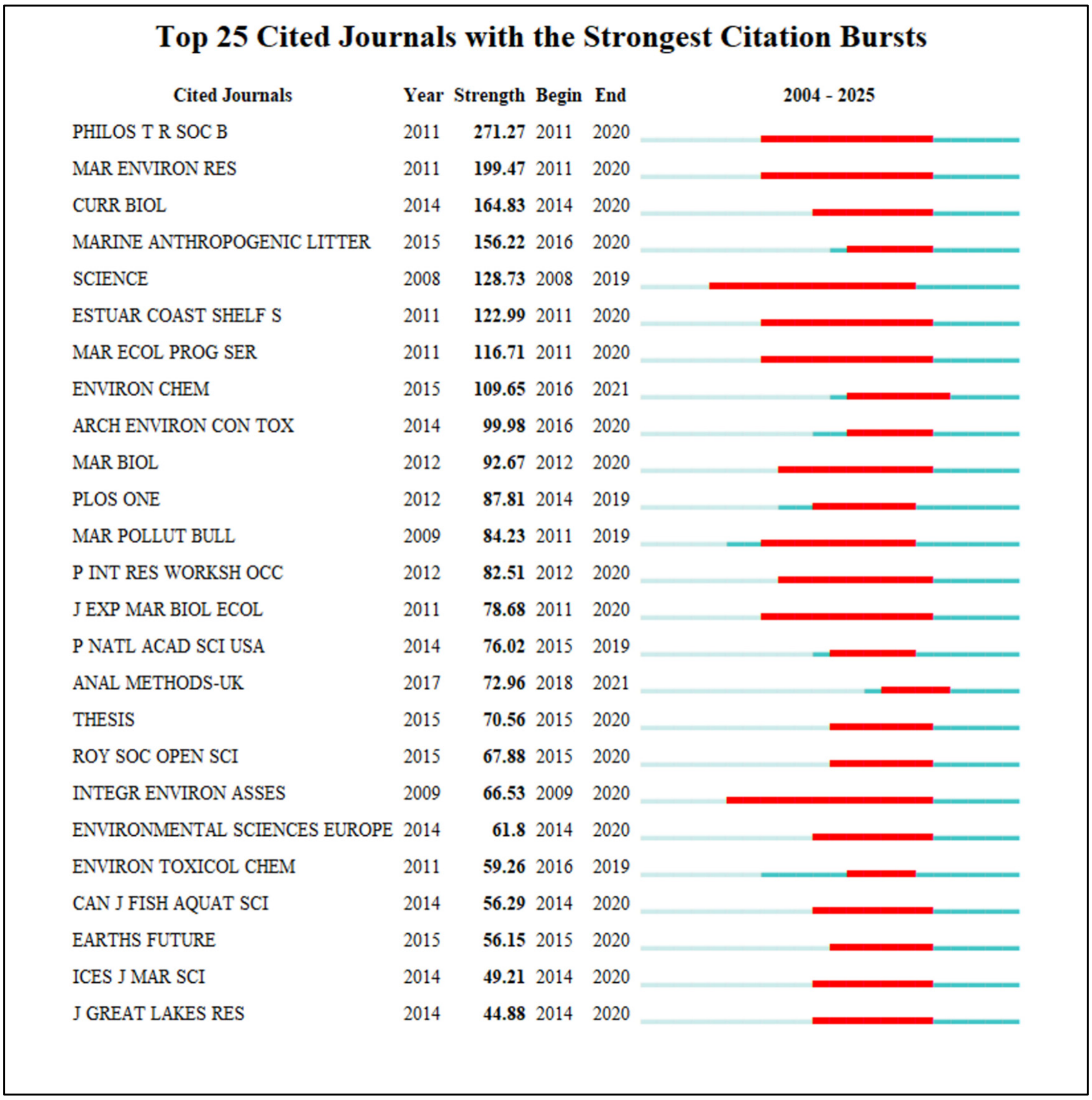

Fig. S12 Top 25 cited journals with the strongest strength of citation bursts (2004-2025).

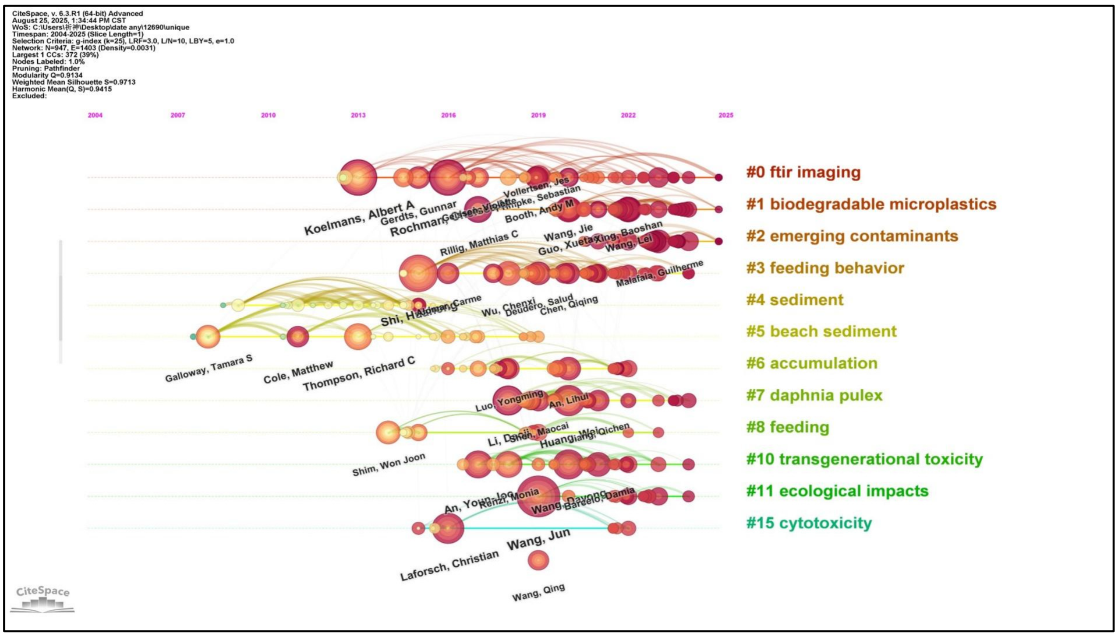

Fig. S13 Timeline visualization of co-occurring author keywords networks (2004–2025)

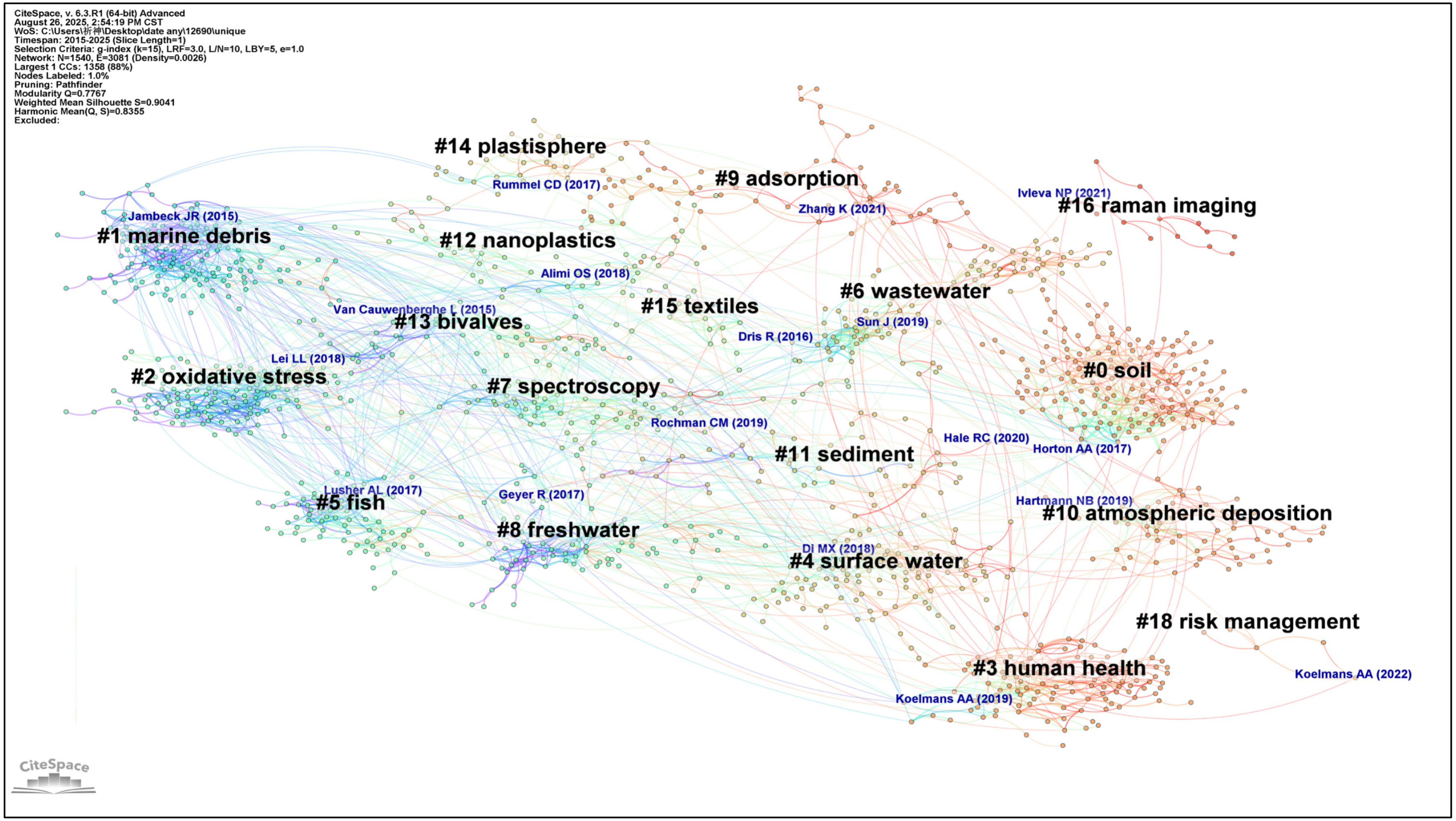

Fig S14 Co-citation references network (2015–2025) and correspondent clustering analysis obtained with CiteSpace

References

Chen, C. 2006. CiteSpace II: Detecting and visualizing emerging trends and transient patterns in scientific literature. Journal of the American Society for Information Science and Technology 57(3), 359-377.

Chen, C., Hu, Z., Liu, S. and Tseng, H. 2012. Emerging trends in regenerative medicine: a scientometric analysis in CiteSpace. Expert Opinion on Biological Therapy 12(5), 593-608.

Chen, C., Ibekwe-SanJuan, F. and Hou, J. 2010. The structure and dynamics of cocitation clusters: A multiple-perspective cocitation analysis. Journal of the American Society for Information Science and Technology 61(7), 1386-1409.

Egghe, L. 2007. Item-time-dependent Lotkaian informetrics and applications to the calculation of the time-dependent h-index and g-index. Mathematical and Computer Modelling 45(7), 864-872.

Freeman, L.C. 1977. A Set of Measures of Centrality Based on Betweenness. Sociometry 40(1), 35-41.

Kleinberg, J. 2003. Bursty and Hierarchical Structure in Streams. Data Mining and Knowledge Discovery 7(4), 373-397.

Mongeon, P. and Paul-Hus, A. 2016. The journal coverage of Web of Science and Scopus: a comparative analysis. Scientometrics 106(1), 213-228.

Newman, M.E.J. 2006. Modularity and community structure in networks. Proceedings of the National Academy of Sciences 103(23), 8577-8582.

Rousseeuw, P.J. 1987. Silhouettes: A graphical aid to the interpretation and validation of cluster analysis. Journal of Computational and Applied Mathematics 20, 53-65.

Sabe, M., Pillinger, T., Kaiser, S., Chen, C., Taipale, H., Tanskanen, A., Tiihonen, J., Leucht, S., Correll, C.U. and Solmi, M. 2022. Half a century of research on antipsychotics and schizophrenia: A scientometric study of hotspots, nodes, bursts, and trends. Neuroscience & Biobehavioral Reviews 136, 104608.

Shibata, N., Kajikawa, Y., Takeda, Y. and Matsushima, K. 2008. Detecting emerging research fronts based on topological measures in citation networks of scientific publications. Technovation 28(11), 758-775.

Stroup, T.S., J, A.L., M, S.S. and McEvoy, J.P. 2000. Comparative effectiveness of antipsychotic drugs in schizophrenia. Dialogues Clin Neurosci 2(4), 373-379.
